# Natural products modulating the hERG channel: heartaches and hope†
†Electronic supplementary information (ESI) available. See DOI: 10.1039/c7np00014f


**DOI:** 10.1039/c7np00014f

**Published:** 2017-05-12

**Authors:** Jadel M. Kratz, Ulrike Grienke, Olaf Scheel, Stefan A. Mann, Judith M. Rollinger

**Affiliations:** a Department of Pharmacognosy , Faculty of Life Sciences , University of Vienna , Althanstraße 14 , 1090 Vienna , Austria . Email: judith.rollinger@univie.ac.at ; Fax: +43-1-4277-855255 ; Tel: +43-1-4277-55255; b CytoBioScience GmbH , Nattermannallee 1 , 50829 Cologne , Germany

## Abstract

Covering: 1996–December 2016

The human Ether-à-go-go Related Gene (hERG) channel is a voltage-gated potassium channel playing an essential role in the normal electrical activity in the heart. It is involved in the repolarization and termination of action potentials in excitable cardiac cells. Mutations in the hERG gene and hERG channel blockage by small molecules are associated with increased risk of fatal arrhythmias. Several drugs have been withdrawn from the market due to hERG channel-related cardiotoxicity. Moreover, as a result of its notorious ligand promiscuity, this ion channel has emerged as an important antitarget in early drug discovery and development. Surprisingly, the hERG channel blocking profile of natural compounds present in frequently consumed botanicals (*i.e.* dietary supplements, spices, and herbal medicinal products) is not routinely assessed. This comprehensive review will address these issues and provide a critical compilation of hERG channel data for isolated natural products and extracts over the past two decades (1996–2016). In addition, the review will provide (i) a solid basis for the molecular understanding of the physiological functions of the hERG channel, (ii) the translational potential of *in vitro*/*in vivo* results to cardiotoxicity in humans, (iii) approaches for the identification of hERG channel blockers from natural sources, (iv) future perspectives for cardiac safety guidelines and their applications within phytopharmaceuticals and dietary supplements, and (v) novel applications of hERG channel modulation (*e.g.* as a drug target).

## Abstract 2

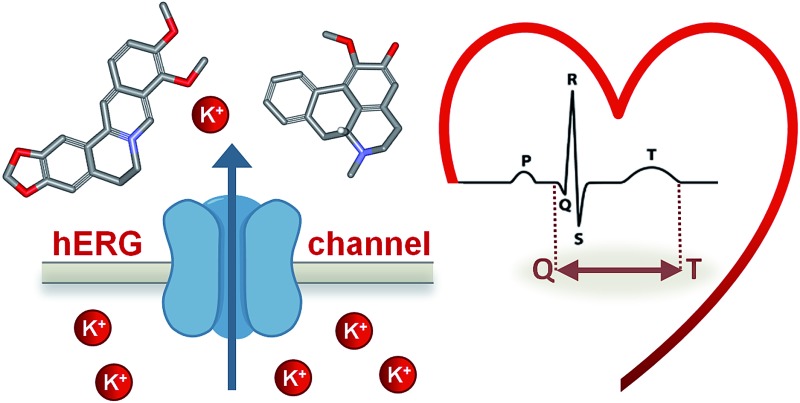
This review covers natural products modulating the hERG potassium channel. Risk assessment strategies, structural features of blockers, and the duality target/antitarget are discussed.

## 1 Introduction

The human Ether-à-go-go Related Gene (hERG) channel controls the efflux of potassium ions from cardiac myocytes. This ion efflux is necessary for the rapid delayed rectifier current during the repolarization phase of the cardiac action potential (AP) and thus crucial for a regular heartbeat.^1^,^2^ An inhibition or blockage of the hERG channel prolongs cardiac repolarization, which is observable as a prolongation of the QT interval on the electrocardiogram (ECG). This blockage can provoke a life-threatening ventricular tachyarrhythmia, so called Torsade de Pointes (TdP), which can lead to sudden death.^2^ Although the mechanisms underpinning drug-induced TdP are far from being fully understood, there are genetic and pharmacological evidences highlighting the pivotal role of the hERG channel. Its blockage is a key factor in pro-arrhythmic liability of a wide range of chemically diverse drugs.^3^–^5^

In the past, severe hERG channel-related cardiac issues led to post-marketing drug withdrawals of non-cardiac drugs, as in the cases of *e.g.* terfenadine (antihistaminic; withdrawn in 1997), sertindole (antipsychotic; withdrawn in 1998), cisapride (gastroprokinetic; withdrawn in 2002), or astemizole (antihistaminic; withdrawn in 2003).^6^–^8^

In 2005, regulatory authorities issued strict preclinical and clinical guidelines aiming at an integrated assessment of the hERG channel-related QT prolonging risk of pharmaceuticals (ICH S7B and E14 Guidelines).^9^,^10^ Implementation of such guidelines successfully prevented the introduction of further torsadogenic drugs into the market by boosting the alertness about this antitarget within early drug discovery and development programs. At the same time, these guidelines also contributed to higher attrition rates through the pipeline.

The emergence of innovative technologies and the vast availability of preclinical and clinical data are now promoting a shift to an improved approach for cardiotoxicity risk assessment, focused on multifactorial torsadogenic mechanisms.^11^,^12^

Despite the awareness of the potential danger of QT prolonging small synthetic molecules, little is known about hERG channel-related cardiotoxicity of medicinal plants and commonly consumed botanicals. This topic is of special relevance since in developing countries, as well as in the industrialized part of the world, herbal remedies are of high importance for the health care system. Herbal preparations continue to increase in popularity, which is also promoted by their over-the-counter (OTC) status, *i.e.* accessibility without prescription of a health care professional. Moreover, natural products are an unquestionable source of structurally diverse chemical scaffolds which are a valuable source for drug discovery. Recently updated statistics by Newman and Cragg underline that 65% of all small molecule approved drugs between 1981 and 2014 can be traced to or were inspired by natural products.^13^ In this perspective, there is an urgent need for studies to critically assess the potential hERG channel-related off-target effects of natural products and to unravel target promiscuity.

This review focuses on natural products modulating the hERG channel, and provides an overview on reported hERG channel interactions taking into consideration the new paradigms of cardiotoxicity assessment. Moreover, the authors discuss recent data supporting the hERG channel as a potential target for drug development, and give an outlook for future prospects.

## 2 The human ether-à-go-go related gene (hERG) channel

### 2.1 Structure of hERG channel and its physiological and pathological roles

The hERG channel, or Kv11.1, is one of several potassium-selective voltage-gated channels that participate in the control of the electrical activity of the human heart.^2^,^5^ The channel is a tetramer, formed by four protein subunits that are trafficked to the outer cellular membrane and spatially arranged to generate a central ion conduction pathway. The protein is encoded by the human ether-à-go-go related gene (or *KCNH2*), and each subunit is formed by six transmembrane segments (S1–S6): S1–S4 form the voltage sensor domain, and S5–S6 form the pore domain together with the loop region. Additionally, NH_2_- and COOH-terminal cytoplasmic domains are present (for a comprehensive review on hERG structure and biology see Vandenberg, J. I. *et al.*).^5^

**Fig. 1:**
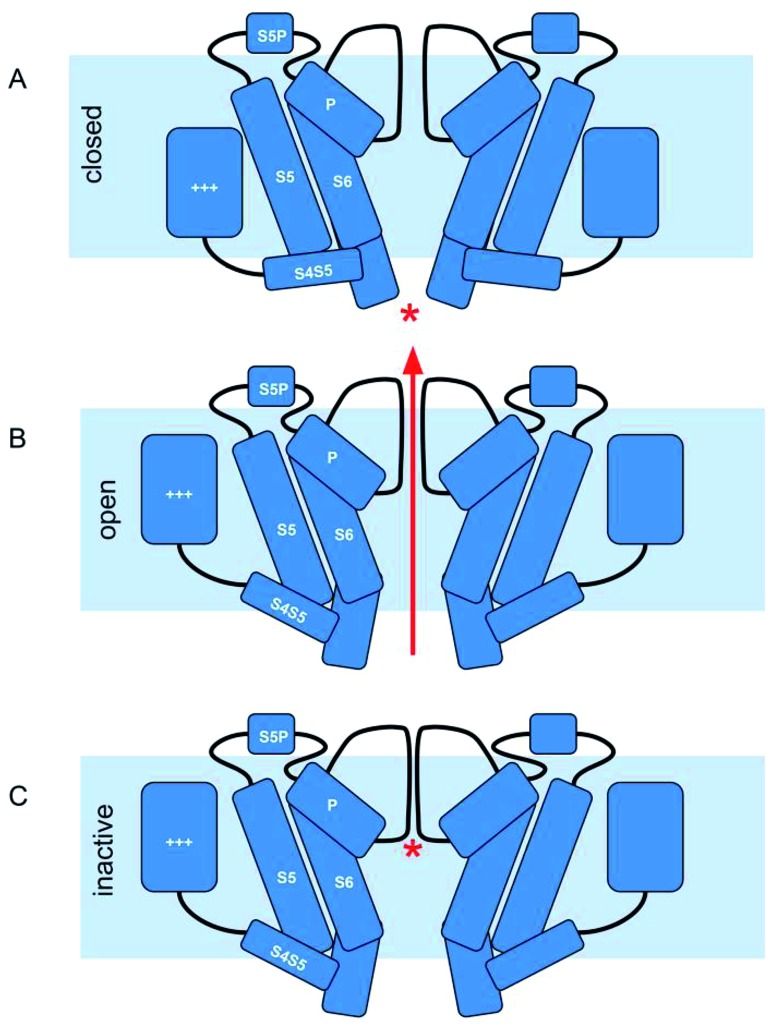
hERG channel gating. (A) At negative membrane potentials hERG channels are in closed state. The voltage sensor domain (+++) is in the down conformation and the cytoplasmic part of the S5/S6 helix bundle prevents the flow of ions (*). (B) Upon depolarization the channels slowly activate: the voltage sensor domain transitions upwards within the membrane. *Via* the S4S5 linker this motion is transferred onto the cytoplasmic end of the helix bundle, and the pore allows potassium ions to flow. (C) The channels rapidly undergo inactivation, which is characterized by a collapse of the selectivity filter (*), preventing any further flow of potassium ions.

Similar to other voltage-gated potassium channels, the hERG channel can be found in three conformational states: closed, open, and inactivated (Fig. 1).

At negative membrane potentials, the channel is in the closed state (S6 helices at the intracellular side bundle together to narrow the pore aperture). Depolarization of the cell membrane induces the opening of the channel, allowing the outward diffusion of potassium ions (S6 helices move apart, increasing the diameter of the pore aperture).

Finally, as the membrane potential progressively moves into depolarization, the channel enters the non-conducting inactivated state. The special features that differentiate the hERG channel from other potassium channels are the kinetics of this process: slow activation and deactivation, coupled to a rapid voltage-dependent inactivation.^2^,^5^

This unusual kinetics allow for the hERG channel to play a key physiological role in the rapid component of the delayed rectified potassium currents (*I*_Kr_) in cardiomyocytes (based on the relative speed of their kinetics, potassium currents are denominated *I*_Kr_ and *I*_Ks_, for rapid and slow components, respectively). These currents are required for the regulation of the duration of the plateau phase during repolarization of the cardiac AP.^2^,^5^

Due to its crucial role in the cardiac AP, impairment of hERG channel function can lead to severe cardiac disorders, manifested by altered QT intervals (the time required for depolarization and repolarization of the ventricles during a single cardiac cycle).^2^ For instance, inherited loss-of-function mutations in hERG can cause long QT syndrome which predisposes individuals to life threatening TdP arrhythmia.^14^ Analogously, inherited gain-of-function mutations are associated with short QT syndrome, a rare genetic anomaly, but also associated with an increased sudden death risk.^15^

### 2.2 Small molecule-induced block of the hERG channel

Even prior to the identification of hERG-related genetic conditions that induce fatal arrhythmias, researchers have already identified that similar effects could be induced by commonly prescribed medications. Indeed, from the conclusive evidence that a large number of small molecules, including several non-cardiac drugs, can block the conduction of potassium ions through the hERG channel, and that this blockage is associated with QT interval prolongation and cardiac liabilities in humans, the hERG channel rose to a priority antitarget. Its inhibition is routinely screened in early drug development, and several drugs have been withdrawn from the market or received restrictions associated with their hERG channel-related cardiotoxicity.^2^,^5^ For a comprehensive review of the history of the hERG channel's role in cardiac risk assessment see Rampe, D. and Brown, A. M.^16^

Perhaps the most striking feature of drug-induced QT prolongation is the structural and therapeutic diversity of hERG channel blockers. No consensus about the binding mode(s) has been reached so far, but combined *in situ* mutagenesis and *in vitro*/*in silico* approaches indicate that most hERG channel blockers interact at the channel cavity, within the pore module.^2^,^17^ Mutation of the S6 aromatic residues Tyr652 and Phe656 substantially decrease the potency of several known blockers, and in addition to their hydrophobic character, these residues allow for a variety of electrostatic interactions with a wide range of chemical substituents.^18^

Analysis of the structures of large sets of hERG channel blockers yielded several structure–activity relationships, and the more general accepted pharmacophore model consists of a basic nitrogen center flanked by aromatic or hydrophobic groups.^19^–^22^ Despite the lack of a crystal structure, several groups postulate that π-stacking interactions between aromatic groups of the blocker and Phe656 and Tyr652 residues of the inner cavity of the hERG channels are required for high-affinity binding, and that the basic nitrogen might form a cation–π interaction with either Tyr652 or Phe656 residues.^2^,^18^

Additionally, there is a growing body of evidence indicating the importance of binding kinetics, and that drugs can interact with the hERG channel in a time-, voltage- and state-dependent manner.^23^,^24^ New hERG-associated mechanisms for acquired long QT syndrome have also been explored, apart from the more well-known inhibition of the hERG channel *via* direct blockage of the pore module. Allosteric modulation^25^ and inhibition of hERG channel trafficking from the cytoplasm to the cellular membrane has already been attributed to several compounds.^16^ These findings will likely improve our overall understanding of the hERG channel, but will also have implications on the preclinical assessment of hERG-related cardiac safety and regulatory perspectives.

### 2.3 Target or antitarget?

In view of the arrhythmogenic risks of drug-induced hERG channel block, this channel has been regarded as an antitarget, and is now an established part of the preclinical cardiotoxicity screening efforts in drug discovery. However, known hERG channel blockers, such as dofetilide, have also been used for many years in the clinic as class III antiarrhythmic agents.^26^ In fact, the potential of the hERG channel as a target is still an open discussion.

New modulators of the channel have been identified during hERG channel block screenings, the so-called activators. These hERG channel agonists enhance potassium currents, and can also pose pro-arrhythmic risks. On the other hand, they also represent therapeutic potential for the treatment of inherited long QT syndrome by shortening the AP duration.^27^ Activators are still relatively rare and little is known about their binding site(s), but most of the molecules studied so far enhanced hERG channel currents mainly by slowing channel deactivation, or by multiple mechanisms.^16^,^27^ Due to pro-arrhythmic safety concerns, the development of hERG channel agonists will likely require the same level of cardiotoxicity assessment as for their blocker counterparts.^16^

Some authors have also postulated the hERG channel might be a promising target in the development of ion channel modulating drugs for the treatment of schizophrenia, cancer, and other co-morbidities.^5^,^28^ The hERG channel is highly expressed in different cell types and tissues (such as the central nervous system and endocrine cells), and frequently upregulated in tumor xenographs and cancer cell lines, being involved in the cell replication cycle.^5^,^28^

The exploration of tissue specific isoforms and advanced drug delivery systems could reduce off-target liabilities and make the development of such drugs feasible.

## 3 Preclinical hERG channel-related cardiotoxicity assessment

### 3.1 *In silico* methods

Great efforts have been applied towards the identification of novel hERG channel blockers and the prediction of cardiac liabilities through computational methods. A comprehensive analysis of this approach is not within the scope of this review (for recent reviews see Jing, Y. *et al.*^29^ and Braga, R. C. *et al.*^20^). In general, these methods can be divided into ligand-based and structure-based approaches; the latter being based on hERG channel homology models.

Ligand-based methods rely on information from the structures of known hERG channel blockers and their popularity reflects the increasing availability of large datasets. Several 2D quantitative structure–activity relationship (QSAR),^30^ 3D QSAR,^31^ 3D pharmacophores,^19^ and classification models^32^ have been published. These models vary on applicability and external validation, but show great potential for early identification of blockers, and can assist lead optimization campaigns by providing valuable information on structure–activity relationships. Notwithstanding, the large chemical diversity of hERG blockers, variability of *in vitro* data, and multiple binding modes and mechanisms of inhibition (*e.g.* trafficking inhibition) impair the development of a global model that performs well over diverse datasets.^33^

Structure-based methods rely on homology models based on the crystal structure of other potassium channels, since a crystal structure of the hERG channel is still not available. Several studies reported the usefulness of these models for the identification of blockers and the study of molecular interactions, state-depended inhibition profiles, and binding modes.^17^,^34^,^35^ Although, the low sequence identity between hERG and the used templates, and the large size and flexibility of the channel pore make it difficult to improve docking predictions.^29^

In summary, multiple *in silico* methods have been developed and, despite their limitations, are highly useful in the early stages of drug discovery. As soon as crystal structures of hERG become available, computational methods will quickly help us to get a better view of the channel structure and to understand the binding dynamics during hERG channel block by small molecules.^36^

### 3.2 *In vitro* assays to detect drug-induced arrhythmia

Preclinical risk assessment of drug-induced arrhythmia has been used by the pharmaceutical industry for decades to determine the pro-arrhythmic liability of new compounds. The same methods can, in principle, be directly applied to natural products and extracts, and are outlined in the following sections.

#### 3.2.1 Cellular assays

The cardiac safety paradigm implemented by the pharmaceutical industry for the last two decades is based on identifying hERG channel liabilities as early as possible, and to restrict structures exhibiting a high efficacy for hERG channel binding and blocking from further development. Most *in vitro* methods which are applied in the early phase of drug safety testing utilize recombinant hERG channel overexpressing cell lines, *i.e.* human embryonic kidney (HEK293) or Chinese hamster ovary (CHO-K1) cells. These preclinical safety assays can be subdivided into high-throughput assays, which allow for the screening of hundreds or even thousands of compounds per day, and lower throughput assays, which typically offer higher data quality than high-throughput methods.

Common high-throughput assays applied in early hERG channel liability screens are voltage-sensitive dye or radioligand binding assays. Both methods do not provide any functional or mechanistic information of how a compound interacts with the hERG channel, and both methods have a low time resolution and do not offer voltage-control, which is of particular disadvantage in case of the hERG channel. Nonetheless, due to their low price per data point they are commonly used prior to the lower throughput automated patch clamp screening.

In fluorescence-based assays, voltage-sensitive dyes are used to monitor changes of the membrane potential in cell lines overexpressing hERG channels. In these cells, the membrane potential is more negative than in untransfected cells, since a small fraction of the channels is in an open, non-inactivated state and permits the efflux of potassium ions. When a test compound blocks hERG channels, the membrane potential becomes more positive. Voltage-sensitive dye-systems report this change in transmembrane voltage either by changes in fluorescence resonance energy transfer (FRET) efficiency or by changes in fluorescence polarization or intensity.^37^,^38^ In response to alterations of the membrane potential, those dyes either relocate from the outside to the inside of the cells (or *vice versa*), leading either to an alteration in fluorescence intensity or a change of the efficiency of FRET with another dye at the outer side of the membrane. In addition to their low sensitivity and their limitation to detect only steady-state effects, interactions of compounds and dyes and compound fluorescence can be an issue in fluorescence assays.

Similar limitations exist for radioligand binding assays. Here, the ability of a compound to displace a radioactively labelled high affinity ligand of the hERG channel, *e.g.*^3^H-dofetilide^39^ or ^3^H-astemizole,^40^ is taken as a readout to estimate the compound's effect on hERG channels. However, in these assays no information is given of how channel function may be altered by the compound, nor whether the compound acts as an agonist or an antagonist on the hERG channel or if there is any state- or use-dependence in channel–compound interaction. Similar to fluorescence assays, the radioligand binding assays provide high throughput and are useful in the early assessment of hERG channel liability. Both, fluorescence and radioligand assays suffer from a high number of false positive and false negative results due to their indirect nature.

**Fig. 2:**
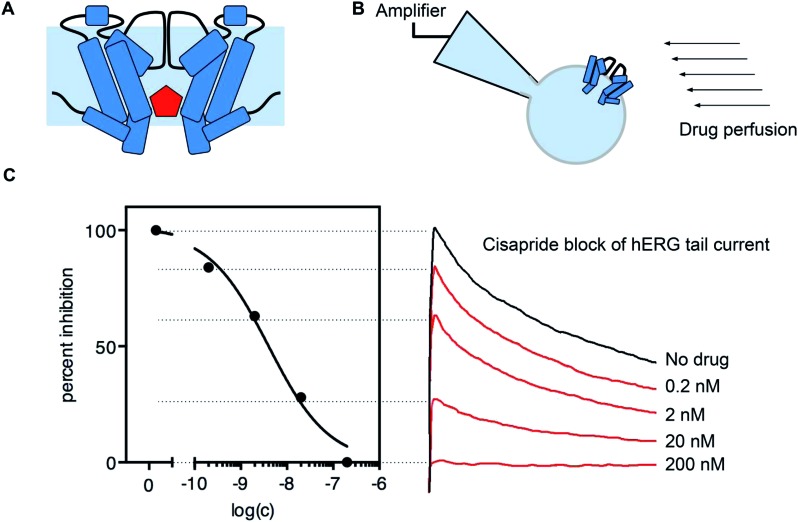
Drug block of hERG channel current. (A) Many drugs (red) can bind in the cavity of the hERG channel and block the flow of potassium ions. (B) Drug block can be measured using the patch clamp method and a perfusion system. (C) Example of hERG channel block by cisapride. Normalized peak tail current amplitudes can be measured and plotted against the applied drug concentration to obtain a dose response curve (data collected with a CytoPatch^4^ Instrument).

The most direct way to measure drug effects on ion channel function is the whole-cell voltage-clamp technique (Fig. 2).^41^*Via* external control of the transmembrane voltage, channel gating can be controlled and the measured ionic current through the ion channels serves as the readout. This method allows for a detailed assessment of how strong a sample blocks hERG channels, and also the mode of action such as state- or use-dependence.

The limitations in throughput and sensitivity, together with the need for qualified (and therefore expensive) lab personnel to perform manual patch clamp measurements, have generated a strong driving force from pharmaceutical companies to automate and upscale the patch clamp technique for the early screening of potential hERG channel liability of new compounds. Over the years, a variety of planar automated patch clamp platforms has emerged and has been utilized for high- and medium-throughput ion channel directed screening. Instead of using glass pipettes, manual micromanipulators and microscopes are used. Patch clamping on these platforms takes place on glass or polymer substrates with one or multiple apertures per recording well or with dedicated pipettes micro-machined into a quartz-glass chip.^42^–^45^

Recently, some devices have been introduced claiming to fulfil both requirements, high-quality data and high-throughput performance. Those devices make use of a 384-well plate format and can generate thousands of datapoints per working day, with a price per data point in the range of 0.15 € (in house data). They are coming close to optical plate readers in terms of productivity and have the potential to replace fluorescence or radioligand binding assays for primary screens.

Besides manual and automated whole-cell voltage-clamp recordings, two electrode voltage-clamp recordings on *Xenopus* oocytes have been reported.^46^ Compared to the patch clamp technique using heterologous expression systems, these recordings are easier to perform and automated systems are available. However, the huge size of the oocytes and the intracellular yolk can cause problems in proper compound application and result in a lower effective intracellular compound concentration compared to HEK293 or CHO cells.^47^

Concerning the assessment of hERG channel liability of plant extracts and mixtures by whole-cell voltage-clamp recordings, the technique to be applied should be carefully considered. Historically, *Xenopus* oocytes have been largely used due to their low costs and straightforwardness. Although, since the number of samples to be tested is relatively low and the physicochemical peculiarities of natural products may be challenging (such as stickiness or solubility issues), it may be worth choosing mammalian cells and manual patch clamp or automated patch clamp devices with a lower throughput, but with high quality voltage-clamp recordings. Additionally, and equally important is to use perfusion systems that minimize compound loss as known from manual patch clamp set-ups.^48^–^50^

#### 3.2.2 Multicellular and organ level assays

The aforementioned hERG channel studies are an important part of cardiac safety pharmacology. However, they do not take into consideration the fact that multiple ion channels, other than hERG, do play a role in the overall pro-arrhythmic risk. Electrical recordings from multicellular preparations, such as Purkinje fibers and papillary muscle from guinea pig, rabbit, or dog, using sharp microelectrodes allow for the measurement of changes in AP parameters after perfusing the cells with the compound of interest.^51^ In particular, any prolongation in the measured action potential duration (APD90, typically) is correlated to QT prolongation in the intact organism. In addition to APD, early after depolarizations (EADs), delayed after depolarizations (DADs), triangulation and reverse use dependence can be assessed in these preparations. The sensitivity of this approach is high, but the reported APD parameter changes depend strongly on species-specific ion channel expression levels and caution must be taken when interpreting results.

Arterially perfused cardiac wedge preparations are more complex, but allow for the simultaneous recording of APD parameters concomitantly with ECG parameters such as velocity of depolarisation through the tissue, or the time it takes for the tissue to repolarise.^52^ They offer high sensitivity as well as high specificity, but the technical challenge and costs associated with these experiments makes them less attractive in the early stages of drug development.

In the most complex *in vitro* assay, the so called Langendorff preparation, rabbit hearts are excised and then retrogradely perfused *via* the aorta.^53^ The excised organ can be kept for several hours in this configuration, and compounds can be added to the perfusate. In addition to ECG parameters, contractile strength can be measured. Langendorff preparations are relatively low in costs, and offer high sensitivity and specificity. They are therefore commonly used in preclinical safety pharmacology. However, like all *in vitro* assays discussed above, they suffer from the fact that neuro-hormonal influences as well as metabolization cannot be tested.

### 3.3 *In vivo* assays to detect drug-induced arrhythmia

To overcome shortcomings of *in vitro* assays, several types of *in vivo* tests have been established. They allow for the assessment of drug metabolism, chronic exposure to drugs, as well as assessment of drug effects on unhealthy hearts (*e.g.* rabbit failing heart). Often these effects are very important, since many drugs have been found to be pro-arrhythmic in structurally or electrically remodeled hearts, but not in the normal population.^54^ Recent technical advances made it possible to record ECG parameters from conscious, freely moving animals such as beagle dogs and rhesus monkeys. These studies have high predictive value, but are expensive to perform and ethical reasons push for a reduction in animal use. Therefore, they are only performed late in the drug development pipeline, before clinical studies commence.^55^

### 3.4 hERG channel block data – comparability and translatability

Apart from the clear applicability of *in vitro* and *in vivo* methods, reported IC_50_ values vary widely between different assay protocols, making it difficult to compare interlaboratorial data. This could be related to a multitude of factors, such as cell line variability, compound stickiness, dose–response calculations, and the so called “run down” effect. For a good discussion on typical pitfalls and artifacts that may lead to misinterpretation of *in vitro* hERG channel block data see Danker, T. and Möller, C.^47^ Additionally, many potent hERG channel blockers display a preference for the inactive state of the channel, *i.e.* they bind with much higher rates to the inactive state of the channel than to the closed or open states. Therefore, the exact choice of pulse protocols and the temperature of the preparation can have profound effects on the reported IC_50_ values for a large number of compounds, as the proportion of time the channel spends in the inactive state largely determines how readily a compound binds and blocks the channel.^56^

Once a drug candidate goes through *in vitro* and *in vivo* scrutiny and clears preclinical safety screening and further development steps, current ICH E14 guidelines suggest a thorough QT study in humans.^10^ These studies are blinded, randomized control studies, performed as early as possible in the clinical phase. Typically, a 30- to 45-fold safety margin of the hERG channel block IC_50_ determined in patch clamp *versus* the free plasma concentration of the drug is widely accepted to provide concordance with clinical QT prolongation and to exclude pro-arrhythmic risk.^3^

However, the predictive value of this systematic approach is still debatable.^57^,^58^ Considering the example of marketed drugs, such as verapamil, ranolazine, and amiodarone, which clearly block the hERG channel and prolong the QT interval in control human subjects, their pro-arrhythmic effect in clinics is notoriously low.^59^,^60^ Undoubtfully, this safety paradigm was successful in a way that drugs such as terfenadine had to be withdrawn from the market and no hERG channel liability was reported to any marketed drug ever since. However, increasing concerns about the relatively low sensitivity of assays and bias toward trappable blockers, highlight the need for improved strategies to strengthen translatability and reduce unwarranted attrition rates, especially during early drug discovery phases where the hERG channel plays a prominent role in go/no-go decisions during candidate selection.^11^,^23^,^58^

### 3.5 Outlook on the evolving torsadogenic risk assessment and novel techniques

To remedy this situation, the comprehensive *in vitro* pro-arrhythmia assay (CiPA) initiative was established in 2013 with the aim to redefine the cardiac safety paradigm.^12^ The CiPA approach is based on the observation that the underlying mechanism of the above-mentioned lack of direct translatability is most likely influenced by the multi ion channel block effect (MICE), where the QT prolonging effect of hERG channel blockade is counteracted by the blockade of depolarizing currents such as L-type calcium channels or voltage-gated sodium channels.^61^ The cardiac AP is shaped by a variety of ion channels and effects of one compound on multiple ion channels may counteract and compensate for each other. It has been shown that correlating the drug efficacy against cardiac ion channels hERG, Nav1.5 and the L-type Ca^2+^ in heterologous expression systems, together with *in silico* integration of cellular electrophysiological effects and fully integrated biological systems (stem-cell-derived cardiomyocytes and human ECG) can give a very good estimate whether a drug may act pro-arrhythmic or not.^11^,^12^,^61^

Within the last few years, the technique of differentiating cardiomyocyte-like cells from induced pluripotent stem cells (iPSC-CM) has made it possible to develop mechanistic medium-throughput cardiac AP assays without the need to sacrifice animals. To test drug effects on cardiac AP different readouts and techniques can be used such as monitoring changes in contractility, or in cell impedance on multi electrode arrays, measure changes in intracellular Ca^2+^ or assess the shape of the AP by utilizing voltage-sensitive dyes. To obtain a full mechanistic understanding of drug effects, a combination of whole-cell voltage-clamp and current-clamp recordings on iPSC-CM would be preferable in order to dissect the drug effects on the single cardiac ionic currents. Additionally, this combination allows to estimate the integrated effect on the cardiac AP. Although no fully automated high-throughput solutions have been demonstrated yet, promising results have been published recently applying iPSC-CM in manual^62^ or automated Gigaseal patch-clamp recordings.^63^ These new technologies are influencing the regulatory authorities and it is very likely that not only manual patch clamp but also automated patch clamp platforms will be accepted for these type of investigations.

In addition, computational models of the human ventricular electrical activity are under development and will be key components of this novel cardiac safety paradigm, aiming at a more mechanistic and reliable assessment of cardiotoxicity risk.^12^ These complex models, based on the O'Hara-Rudy model of cardiac action potentials,^64^ integrate electrophysiology data on multiple ion channels, and consider the effects of time-, voltage- and state-dependent changes in drug–hERG channel interaction.^12^,^64^,^65^

## 4 Approaches for the identification of hERG channel blockers of natural origin

As opposed to the search for beneficial bioactivities of natural materials, the screening of plant extracts for interactions with the hERG channel is often neglected or not pursued. In the context of drug discovery from nature, an extract that has been identified to contain hERG blocking constituents becomes rather unattractive for further isolation processes since its potential for the identification of drug leads decreases tremendously. In the scientific literature, these “undesired” results might also be difficult to access due to the lack of open access databases and the narrow possibilities for publishing such findings. So far, only a few natural product research groups have accordingly focused on the identification of hERG channel blocking natural compounds. Moreover, in phytochemistry and natural product drug discovery researchers are often not aware of possible off-target or antitarget effects such as cardiotoxicity *e.g.* caused by the blockage of the hERG channel.

To address these issues, the EU Marie Curie FP7 PEOPLE IRSES project “hERG related risk assessment of botanicals” (hERGscreen, P295174) was launched in 2012. The main goal of this project was the establishment of a network between European, South American, North American, and South African educational and research entities to focus on the identification of hERG channel blockers in commonly consumed botanicals and supplements.

For the target-oriented identification and isolation of hERG channel blocking constituents from natural sources, three different strategies have been applied: (i) *in vitro* screening of plant extracts and commonly consumed herbal preparations followed by microfractionation, activity profiling, and isolation, (ii) investigation of plant materials based on reports of cardiotoxicity without a clear understanding of the molecular mechanism, and (iii) computational approaches (*e.g.* virtual prediction of hERG channel blockage). Moreover, the combination of these techniques has proven to speed up the evaluation of hERG channel-related safety aspects of natural products.

### 4.1 *In vitro* screening of extracts and complex mixtures

hERG channel interactions of natural compounds are primarily reported by pharmacology research groups who tend to focus on the blockage mechanism of a single compound and its kinetics rather than on the activity assessment of multicomponent extracts.

However, since 2011 there is an increase of publications reporting on hERG channel blocking extracts, which correlates with the launch of the hERGscreen project. In this project, an international multidisciplinary research consortium worked on the investigation of the hERG channel blocking potential of >1200 extracts of herbal drugs of the European and Chinese Pharmacopoeias as well as edible plants and widely used herbal medicines from South America, Africa, and Europe.^48^,^49^,^66^,^67^,^69^ Plant materials were selected based on the reported content of alkaloids, their high medicinal relevance, and their common use. Primarily, extracts were tested at 100 μg mL^–1^ in the voltage-clamp assay using *Xenopus* oocytes and a reduction of the hERG peak tail current by ≥30% was considered as selection criterion for closer investigations (Table 1).

**Table 1:** Overview on plant extracts showing hERG blockage at 100 μg mL^–1^ in the voltage-clamp assay using *Xenopus* oocytes^*a*^

Plant species	Family	Plant organ	Type of extract	hERG channel inhibition [%]	Ref.
*Carapichea ipecacuanha* (Brot.) L. Andersson	Rubiaceae	Roots & rhizomes	LLE	32.5	49
*Chelidonium majus* L.	Papaveraceae	Herb	LLE	47.9	49
*Cinchona pubescens* Vahl	Rubiaceae	Bark	LLE	45.3	49
*Cinnamomum zeylanicum* Nees	Lauraceae	Bark	MeOH	64.5	66
*Coptis chinensis* Franch.	Ranunculaceae	Rhizomes	MeOH	31.7	67
*Evodia rutaecarpa* (Juss.) Hook. f. et Thoms.	Rutaceae	Fruits	MeOH	60.9	68
*Galenia africana* L.	Aizoaceae	Stems & leaves	CH_2_Cl_2_	50.4	69
*Gnidia polycephala* Gilg ex Engl.	Thymelaeaceae	Roots	CH_2_Cl_2_	58.8	70
*Myristica fragrans* L.	Myristicaceae	Seeds	MeOH	42.3	66
*Nelumbo nucifera* Gaertn.	Nelumbonaceae	Leaves	Alkaloid fraction	50.4	48 and 49
*Paullinia cupana* Kunth	Sapindaceae	Seeds	MeOH	45.3	66
*Piper nigrum* L.	Piperaceae	Fruits	EtOAc	32.4	66
			MeOH	36.9	
*Rauwolfia serpentina* L.	Apocynaceae	Roots	LLE	39.1	49

^*a*^LLE – lead-like enhanced extracts;^49^,^71^ MeOH – methanol; CH_2_Cl_2_ – dichloromethane; EtOAc – ethyl acetate.

Several lead-like enhanced extracts^49^,^71^ showed a strong inhibition of the hERG channel current, with the extracts of ipecac (*Carapichea ipecacuanha* (Brot.) L. Andersson; roots and rhizomes), greater celandine (*Chelidonium majus* L.; herb), the quinine tree (*Cinchona pubescens* Vahl; bark), lotus (*Nelumbo nucifera* Gaertn.; leaves), and Indian snakeroot (*Rauwolfia serpentina* L.; roots) being prominent examples, inhibiting the hERG channel current by 32.5%, 47.9%, 45.3%, 50.4%, and 39.1%, respectively.^48^,^49^

Regarding African plants, dichloromethane extracts from the stems and leaves of *Galenia africana* L. and the roots of *Gnidia polycephala* Gilg ex Engl. reduced the hERG channel current by 50.4% and 58.8%, respectively.^69^,^70^ From the latter plant material, it is the first time that diterpenes – in this case daphnane-type diterpenoid orthoesters (**1–3**) – were identified as hERG channel blockers.
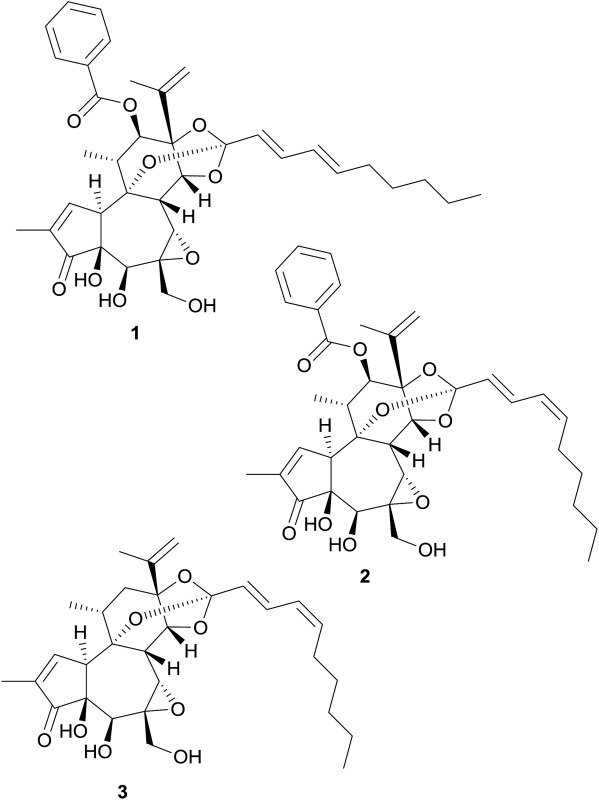


Another prominent example for a hERG channel blocking extract is the methanol extract of the rhizomes of *Coptis chinensis* Franch. which reduced the hERG channel peak tail current by 31.7%.^67^ Subsequently, this effect could be successfully narrowed down to dihydroberberine (**4**) as the responsible constituent (30.1% reduction of hERG channel current at 100 μM).^67^
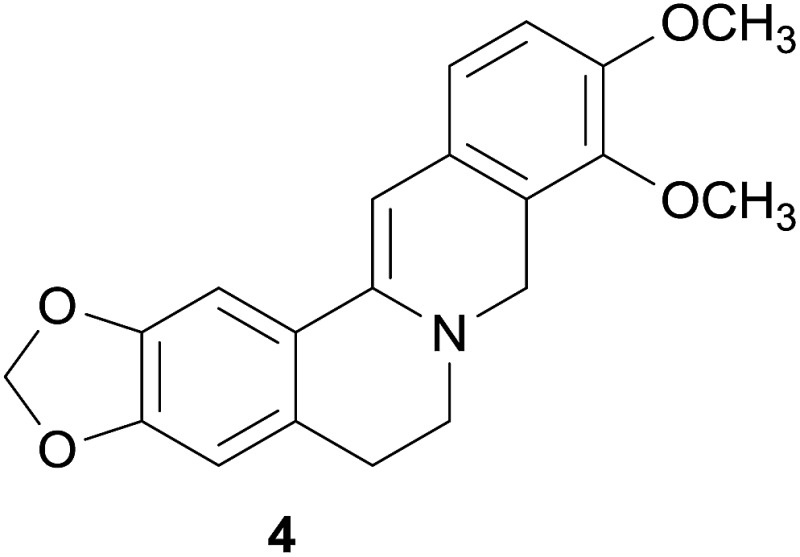


Further methanol extracts of the common spices cinnamon (*Cinnamomum zeylanicum* Nees; bark), guarana (*Paullinia cupana* Kunth; seeds), nutmeg (*Myristica fragrans* L.; seeds), and the ethyl acetate and methanol extracts of the fruits of black pepper (*Piper nigrum* L.) were found to be potential hERG channel blocking multicomponent mixtures, which inhibited the hERG channel current by 64.5%, 45.3%, 42.3%, 32.4%, and 36.9%, respectively.^66^ However, the observed effects for cinnamon, guarana, and nutmeg were possibly related to the presence of tannins, since tannin-depleted extracts did not lead to such high percentages of hERG channel blockage. Due to their low oral bioavailability and thus a negligible hERG channel-related risk for consumers, these extracts were not further pursued. Concerning the extracts of black pepper fruits, the hERG channel current reducing activity could be traced down to the major constituent piperine (**5**) by means of activity profiling of microfractions (see 4.2).^66^
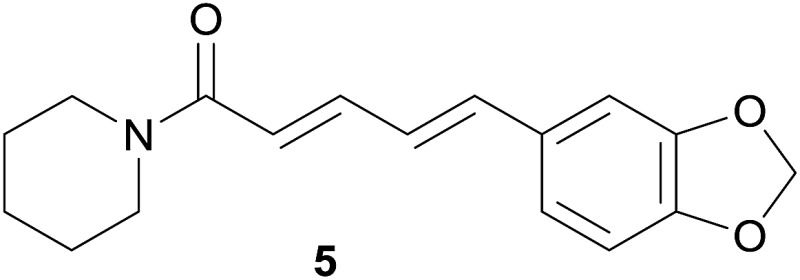


Among further extracts tested, the methanol extract of the fruits of *Evodia rutaecarpa* (Juss.) Hook. f. et Thoms. reduced the peak tail hERG channel current by 60.9%. Two indoloquinazoline alkaloids, *i.e.* dehydroevodiamine (**6**) and hortiamine (**7**), were identified as the active principles.^68^
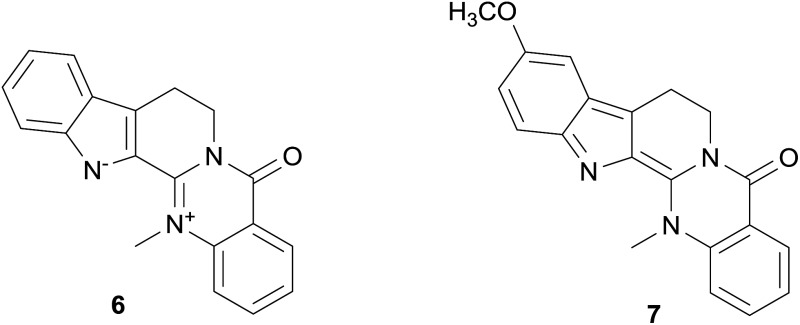


Besides the “hERGscreen” consortium, also the Hungarian research group around J. Hohmann investigated the hERG channel blocking effects of several plant extracts in an electrophysiological study. For instance, hydroethanolic extracts of greater celandine (*Chelidonium majus* L.; herb), a plant material which is officinal in the European Pharmacopoeia, were tested in a patch-clamp assay using HEK293 cells.^72^ These orally used herbal preparations produced with 25% and 45% ethanol significantly inhibited the hERG channel with estimated IC_50_s of 8.30 μg mL^–1^ and 5.09 μg mL^–1^, respectively.^72^ Following up on these effects, alkaloids present in the extracts were found to contribute to the observed inhibition. Moreover, the greater celandine extracts (*c* = 5 μg mL^–1^) were found to moderately prolong the AP duration in a microelectrode assay using dog ventricular muscle preparations.

### 4.2 Microfractionation and activity profiling

**Fig. 3:**
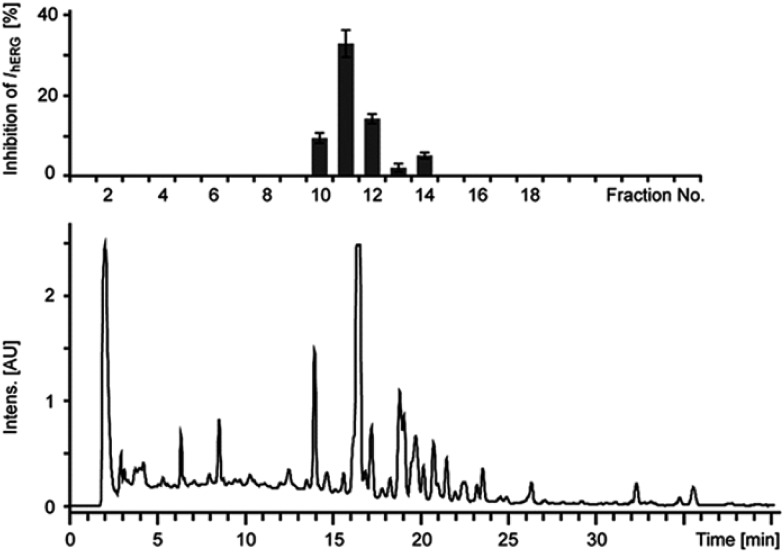
Microfractionation and activity profile of an extract with HPLC-PDA chromatogram (lower trace, at 254 nm) and corresponding activity profile (upper trace, % inhibition of hERG channel current) of time-based microfractions. Reproduced from reference Schramm, A. *et al.*^66^ with permission from Georg Thieme Verlag, Stuttgart.

For the risk assessment of frequently consumed botanicals the method of combining HPLC-microfractionation with on-line and off-line spectroscopy and subsequent activity profiling, as reported by the group of M. Hamburger, has evolved as promising and well-focused strategy.^67^,^73^,^74^ Here, microfractionation of an extract includes the separation into time-based fractions with the help of a semi-preparative HPLC instrument and the processing of these microfractions for *in vitro* testing on hERG channel blockage (Fig. 3). For this procedure extracts are usually dissolved in DMSO or DMSO/MeOH mixtures at a concentration of 100 mg mL^–1^. For separation, the dissolved extracts are then applied onto a semi-preparative RP-HPLC column. By using a previously optimized HPLC system, the extract is fractionated based on time, *i.e.* usually 90 s per fraction. Under reduced pressure the solvent is then removed from the obtained fractions.

In order to avoid false positive results from tannin interfering extracts in the *in vitro* test system, the removal of tannins prior to extract screening and microfractionation is highly recommended. A very useful protocol for the generation of tannin-depleted extracts further enriched with orally bioavailable constituents (so called lead-like enhanced extracts) has been described recently.^49^,^71^

### 4.3 Multidisciplinary approaches

Computer aided screening technologies can be applied to identify novel lead structures from in house or commercially available natural compound databases, and for the targeted selection of botanicals potentially blocking the hERG channel. Using virtual screenings in combination with *in vitro* screenings has proven to provide significantly higher enrichments of active molecules than performing high-throughput screening solely.^75^,^76^

Surprisingly, this approach has been scarcely applied for hERG channel screening of natural compounds. Within the “hERGscreen” project, a previously validated pharmacophore model^19^ was applied for the virtual screening of a 3D multiconformational database consisting of 125 723 natural products. From the virtual hitlists it was possible to trace back plant species containing putative hERG channel blockers, and prioritize them for *in vitro* testing according to their medicinal relevance. The *in vitro* screening of these plant extracts showed a high percentage of true blockers, including unreported blockers, and thus corroborating the applicability of this multidisciplinary approach.^49^

On the other hand, the computational-based strategy also presents drawbacks since the performance of *in silico* models can drop significantly when applied to chemical space outside of that defined by the original training sets or to chemically diverse databases focused on natural products.^19^,^33^ For instance, we virtually screened the database of natural compounds compiled for this review with one of the validated pharmacophore models generated within the “hERGscreen” project,^19^ and the QSAR-based Pred-hERG online tool.^31^ The results from this virtual campaign showed both approaches could identify roughly one third of the strong blockers (36% and 39%, respectively), but performed much better with the non-blockers (both methods with ≥90% true negatives).

## 5 hERG channel blockers of natural origin

With around ∼300 000 plant species and ∼600 000 fungi and microbial organisms, nature offers a huge biological and chemical diversity.^77^ Today, even after the impact of combinatorial chemistry in the late 1980s, nature is still an interesting source to search for drug lead scaffolds.^13^,^78^ In this continuing endeavor, safety aspects in the context of antitarget or off-target effects should not be neglected. Hence, it is of high importance to collect and critically evaluate all available data on natural product hERG blockers.

Regarding hERG channel-related risks of natural compounds, it needs to be emphasized that all available *in vitro* data irrespective of the potency, *i.e.* strong, moderate, or no blockage, have been considered for this review. This gives a comprehensive picture of the current state of research and listing also non-blockers is of particular relevance to avoid retesting, to deduce important structural information, *e.g.* for *in silico* models, and to gain insights into structural requirements for blockers *vs.* non-blockers.

For data collection concerning natural compounds (Table 2) the two most common assay systems were selected, *i.e.* the whole-cell patch-clamp assay (either using HEK293 cells or CHO-K1 cells) and the two-microelectrode voltage-clamp assay using *Xenopus* oocytes. To allow for a categorization of the collected data, a threshold between strong, moderate, and non-blocker was set. Due to the different sensitivity of applied test systems, compounds with IC_50_ values ≥100 μM obtained by the patch-clamp as well as voltage-clamp system or <25% hERG blockage at 100 μM in the voltage-clamp system were considered as non-blockers. Compounds with IC_50_s of >10 to <100 μM (patch-clamp system) or IC_50_s of >25 to <100 μM (voltage-clamp system) were classified as moderate blockers. Accordingly, strong hERG blockers are characterized by IC_50_ values ≤10 μM (patch-clamp system) or IC_50_s ≤ 25 μM (voltage-clamp system).

**Table 2:** Overview on natural compounds tested for hERG channel inhibition (alphabetically listed)^*a*^

Name	No.	Natural source* species (family)	hERG channel inhibition			Ref.
			Category	IC_50_ [μM]*	Assay type	
**Alkaloids**						
Aconitine	**37**	*Aconitum* sp. (Ranunculaceae)	+		B	80 and 106
			++	1.8	C	107
			++	13.5	D	108
Aconosine	**38**	*Aconitum anthora* L. (Ranunculaceae)			B	80 and 106
Acotoxicine	**39**	*Aconitum anthora* L. (Ranunculaceae)	–		B	80 and 106
Acotoxinine	**40**	*Aconitum anthora* L. (Ranunculaceae)	–		B	80
Acovulparine	**41**	*Aconitum anthora* L. (Ranunculaceae)	–		B	80
Ajacine	**42**	*Aconitum anthora* L. (Ranunculaceae)	–		B	80
Ajmaline	**43**		++	1.0	A	109
			+	42.3	C	109
Allocryptopine	**44**	*Corydalis decumbens* (Thunb.) pers. (Papaveraceae)	+	49.7	A	110
Arecoline	**45**		++	9.6	A	111
(–)-Argemonine	**46**		–		C	112
(–)-Asimilobine	**14**	*Nelumbo nucifera* Gaertn. (Nelumbonaceae)	–		C	48
Azaspiracid 1	**47**	*Azadinium* sp. (Dinophyceae)	++	0.8	A	113
Azaspiracid 2	**48**	*Azadinium* sp. (Dinophyceae)	++	0.6	A	113
Azaspiracid 3	**49**	*Azadinium* sp. (Dinophyceae)	++	0.8	A	113
14-Benzoylaconine 8-*O*-palmitate	**50**	*Aconitum anthora* L. (Ranunculaceae)	+		B	80
Benzoylecgonine	**16**	Metabolite of cocaine	–		A	114
Benzyltetrahydropalmatine	**51**	*Corydalis ambigua* Cham. & Schltdl. (Papaveraceae)	+	22.4	A	110
Berberine	**10**	*Berberis* sp. & *Coptis* sp. (Berberidaceae; Ranunculaceae)	++	3.1	A	115
			++	6.5	A	72
			+	80	C	115
			+	∼75	C	67 and 116
Bisindolylmaleimide I	**52**		++	0.8	A	117
(+)-Boldine	**53**		++	19.3	C	112
(+)-Bulbocapnine	**54**		++	7.4	C	112
Caffeine	**28**		–		A	118
(–)-Californidine	**55**		–		C	112
Capsaicin	**56**	*Capsicum* sp. (Solanaceae)	++	17.5	C	119
Cephaeline	**57**	*Carapichea ipecacuanha* (Brot.) L. Andersson (Rubiaceae)	++	5.3	A	49
Changrolin	**58**	*Dichroa febrifuga* Lour (Hydrangeaceae)	+	18.2	A	120
Chelerythrine	**59**		++	0.1 (EC_50_)	A	117
Chelidonine	**60**	*Chelidonium majus* L. (Papaveraceae)	++	1.0	A	72
			++	11.5	C	112
Cocaethylene	**17**	Metabolite of cocaine	++	1.2	A	114
Cocaine	**61**		++	7.2	A	121
			++	4.4	A	114
			++	8.7–14.4	A	122
			++	∼4	D	123
			++	5.6	D	124
Codeine	**26**		–	>300	A	91
			–	97	B	92
Coptisine	**12**	*Chelidonium majus* L., *Coptis chinensis* Franch., (Papaveraceae; Ranunculaceae)	+	90.1	A	72
			–		C	67
(+)-Corynoline	**62**		++	7.1	C	112
Cyclovirobuxine D	**63**	*Buxus microphylla* Siebold & Zucc. (Buxaceae)	+	19.7	A	125
Dauricine	**64**	*Menispermum dauricum* DC. (Menispermaceae)	++	3.5	A	126
			+	16–33	D	127
Daurisoline	**65**	*Menispermum dauricum* DC. (Menispermaceae)	++	9.1/9.6	A	128
Delcosine	**66**	*Aconitum anthora* L. (Ranunculaceae)	–		B	80
Delectinine	**67**	*Aconitum anthora* L. (Ranunculaceae)	–		B	80 and 106
14-Desacetyl-18-demethylpubescenine	**68**	*Aconitum anthora* L. (Ranunculaceae)	–		B	80 and 106
Dehydroevodiamine	**6**	*Evodia rutaecarpa* (Juss.) Hook. f. et Thoms. (Rutaceae)	++		C	68
Dihydroberberine	**4**	*Coptis chinensis* Franch. (Ranunculaceae)	+		C	66 and 67
Dihydrosanguinarine	**69**		–		C	112
Dolaconine	**70**	*Aconitum anthora* L. (Ranunculaceae)	–		B	80 and 106
Ecgonine methylester	**71**	Metabolite of cocaine	–		A	114
Emetine	**72**	*Carapichea ipecacuanha* (Brot.) L. Andersson (Rubiaceae)	+	21.4	A	49
			–		C	112
Ephedrine	**73**		–		A	129
			–		C	112
Epiberberine	**74**	*Coptis chinensis* Franch. (Ranunculaceae)	–		C	67
(–)-Eschscholtzine	**75**		–		C	112
Flavopereirine	**76**	*Rauwolfia nukuhivensis* (Fosberg & Sachet) Lorence & Butaud (Apocynaceae)	+		B	81
Galanthamine	**77**		–	760	A	130
			–		C	112
			–		D	130
(–)-Galanthine	**78**		–		C	112
Gigactonine	**79**	*Aconitum anthora* L. (Ranunculaceae)	+		B	80
Guan-fu base A	**80**	*Aconitum coreanum* (H.Lév.) Rapaics (Ranunculaceae)	–	1640	A	131
Guan-fu base G	**81**	*Aconitum coreanum* (H.Lév.) Rapaics (Ranunculaceae)	+	17.9	A	131
(+)-Haemanthamine	**82**		–		C	112
Harmine	**83**		+		C	112
Hetisinone	**84**	*Aconitum anthora* L. (Ranunculaceae)	–		B	80
(+)-Hippeastidine	**85**		+		C	112
Hortiamine	**7**	*Evodia rutaecarpa* (Juss.) Hook. f. et Thoms. (Rutaceae)	++		C	68
10-Hydroxy-8-*O*-methyltalatizamine	**86**	*Aconitum anthora* L. (Ranunculaceae)	–		B	80
Ibogaine	**87**	*Tabernanthe iboga* Baill. (Apocynaceae)	++	3.53	A	132
Indirubin	**88**		–		C	112
Isotalatizidine	**89**	*Aconitum anthora* L. (Ranunculaceae)	–		B	80
Jatrorrhizine	**90**	*Coptis chinensis* Franch. (Ranunculaceae)	–		C	67
Karacoline	**91**	*Aconitum napellus* subsp. Firmum (Rchb.) Gáyer (Ranunculaceae)	–		A	106
Liensinine	**92**	*Nelumbo nucifera* Gaertn. (Nelumbonaceae)	–		A	48 and 133
Lobeline	**93**	*Lobelia inflata* L. (Campanulaceae)	++	0.3	A	134
Lochneram	**94**	*Rauwolfia nukuhivensis* (Fosberg & Sachet) Lorence & Butaud (Apocynaceae)	+		B	81
Lycoctonine	**95**	*Aconitum anthora* L. (Ranunculaceae)	–		B	80
Matrine	**20**	*Sophora flavescens* Aiton (Fabaceae)	–		A	89
			–	411	B	135
18-Methoxycoronaridine	**96**	Semisynthetic	+	>50	A	132
10-Methoxypanarine	**97**	*Rauwolfia nukuhivensis* (Fosberg & Sachet) Lorence & Butaud (Apocynaceae)	+		B	81
Methylecgonidine	**98**	Metabolite of cocaine	–	171.7	A	114
*N*-Methylemetine	**99**	*Carapichea ipecacuanha* (Brot.) L. Andersson (Rubiaceae)	–		A	49
(+)-*N*-Methyllaurotetanine	**100**		++	3.4	C	112
*N*_12_-Methylnukuhivensium	**101**	*Rauwolfia nukuhivensis* (Fosberg & Sachet) Lorence & Butaud (Apocynaceae)	++	0.4	B	81
*O*-Methylpsychotrine	**102**	*Carapichea ipecacuanha* (Brot.) L. Andersson (Rubiaceae)	+	12.5	A	49
Morphine	**25**		–	>1000	A	91
Napelline	**103**	*Aconitum napellus* subsp. Firmum (Rchb.) Gáyer (Ranunculaceae)	–		A	106
Neferine	**104**	*Nelumbo nucifera* Gaertn. (Nelumbonaceae)	++	7.4	A	133 and 136
(–)-Neocaryachine	**105**		–		C	112
Neoline	**106**	*Aconitum anthora* L. (Ranunculaceae)	–		B	80 and 106
Neolinine	**107**	*Aconitum anthora* L. (Ranunculaceae)	+		B	80 and 106
Nicotine	**108**		++	16.8	C	137
			++		D	138
			++	1.3	D	139
Noribogaine	**109**	Metabolite of ibogaine	++	2.9	A	132
*N*-Nornuciferine	**110**	*Nelumbo nucifera* Gaertn. (Nelumbonaceae)	++	9.8	A	48
*O*-Nornuciferine	**15**	*Nelumbo nucifera* Gaertn. (Nelumbonaceae)	++	7.9	A	48
Norsandwicine	**111**	*Rauwolfia nukuhivensis* (Fosberg & Sachet) Lorence & Butaud (Apocynaceae)	+		B	81
Nortueiaoine	**112**	*Rauwolfia nukuhivensis* (Fosberg & Sachet) Lorence & Butaud (Apocynaceae)	+		B	81
Noscapine	**113**		–		C	112
Nuciferine	**114**	*Nelumbo nucifera* Gaertn. (Nelumbonaceae)	++	2.9	A	48
Nukuhivensium	**115**	*Rauwolfia nukuhivensis* (Fosberg & Sachet) Lorence & Butaud (Apocynaceae)	++	4	B	81
8-Oxoberberine	**11**		–		C	112
8-Oxocoptisine	**13**		–		C	112
Oxymatrine	**21**	*Sophora flavescens* Aiton (Fabaceae)	–	665	A	90
			–		B	89
Palmatine	**116**	*Coptis chinensis* Franch. (Ranunculaceae)	+		C	67
Papaverine	**117**	*Papaver somniferum* L. (Papaveraceae)	++	7.3	A	129
			++	0.6	A	140
			+	71.0	C	141
			+	30.0	C	140
Piperine	**5**	*Piper nigrum* L. (Piperaceae)	–	>300	C	66
Protopine	**118**	*Eschscholzia californica* Cham. (Papaveraceae)	++	4.1	C	112
Psychotrine	**119**	*Carapichea ipecacuanha* (Brot.) L. Andersson (Rubiaceae)	–		C	49
Pyroaconitine	**120**	*Aconitum anthora* L. (Ranunculaceae)	–		B	80
Quinidine	**9**		++	0.4	A	142
			++	3.2	B	143
			++	4.6	C	85
			++	0.7	D	108
Quinine	**8**		+	57.0	C	85
(–)-Remerine	**121**		+		C	112
Reserpine	**122**		++	1.9	B	144
			++	7.6	C	112
			++	4.9	D	144
(+)-Reticuline	**123**		+		C	112
Rhynchophylline	**124**	*Uncaria rhynchophylla* (Miq.) Miq. ex Havil. (Rubiaceae)	–	773	C	145
(+)-Salutaridine	**27**		–		C	112
Sandwicine	**125**	*Rauwolfia nukuhivensis* (Fosberg & Sachet) Lorence & Butaud (Apocynaceae)	+		B	81
Sanguinarine	**126**	*Chelidonium majus* L. (Papaveraceae)	++	0.9	A	72
Senbusine A	**127**	*Aconitum napellus* subsp. Firmum (Rchb.) Gáyer (Ranunculaceae)	–		A	106
Senbusine C	**128**	*Aconitum napellus* subsp. Firmum (Rchb.) Gáyer (Ranunculaceae)	–		A	106
Septentriodine	**129**	*Aconitum anthora* L. (Ranunculaceae)	–		B	80
Songoramine	**130**	*Aconitum anthora* L. (Ranunculaceae)	+		B	80
Songorine	**131**	*Aconitum anthora* L. (Ranunculaceae)	–		B	80 and 106
Sophocarpine	**18**	*Sophora flavescens* Aiton (Fabaceae)	–	100–300	A	88
Sophoridine	**19**	*Sophora flavescens* Aiton (Fabaceae)	–	>300	A	87
(–)-Sparteine	**132**		–		C	112
Spegatrine	**133**	*Rauwolfia nukuhivensis* (Fosberg & Sachet) Lorence & Butaud (Apocynaceae)	+		B	81
Swatinine	**134**	*Aconitum anthora* L. (Ranunculaceae)	–		B	80
Takaosamine	**135**	*Aconitum anthora* L. (Ranunculaceae)	–		B	80
(+)-Tazettine	**136**		–		C	112
Tetrahydroberberine	**137**	*Coptis chinensis* Franch. (Ranunculaceae)	–		C	67
Theobromine	**30**		–		B	92
Theophylline	**29**		–		C	141
Tryptanthrin	**24**		–		C	112
Vasicine	**22**		–		C	112
Vasicinone	**23**		–		C	112
Voacangine	**138**	Metabolite of ibogaine	++	0.3/2.3	A	19 and 132
Yohimbine	**139**		+	67.1	C	112

**Non-alkaloids**						
Acacetin	**140**	*Saussurea laniceps* Hand.-Mazz. (Asteraceae)	+	32.4	A	146
Allitridin	**141**	*Allium sativum* L. (Amaryllidaceae)	+	19.6	A	147 and 148
Apigenin	**142**		–		C	93
Bergapten	**143**		–		C	93
(+)-Catechin	**144**		–		C	66
Celastrol	**145**		++		A	149
Chrysin	**146**		–		C	93
Coumarin	**147**		–		C	93
Curcumin	**34**		++	5.6/4.9	A	99–101
			+	22	B	144
Digitoxin	**148**		–		A	150
Digoxigenin	**149**		–		A	150
Digoxin	**150**		–		A	150
Dihydroartemisinin	**151**		++	9.6	A	151
5,7-Dihydroxy-6-methoxyflavone	**152**	*Galenia africana* L. (Aizoaceae)	–		C	69
(2*R*)-6,7-Dimethoxyflavanone	**153**	*Galenia africana* L. (Aizoaceae)	–		C	69
6,7-Dimethoxyflavone	**154**	*Galenia africana* L. (Aizoaceae)	–		C	69
7,8-Dimethoxyflavone	**155**	*Galenia africana* L. (Aizoaceae)	–		C	69
EGCG	**33**	*Camellia sinensis* (L.) Kuntze (Theaceae)	++	6.0	A	97
			–		B	98
			++	20.5	C	97
Ellagic acid	**156**		–		C	66
7-Ethoxycoumarin	**157**		–		C	93
(4′*Z*)-Excoecariatoxin	**3**	*Gnidia polycephala* Gilg ex Engl. (Thymelaeaceae)	++		C	70
Fisetin	**158**		–		C	93
Flavone	**159**		–		C	93
Galangin	**160**		–		C	93
Gallic acid	**161**		–		C	66
Ginsenoside Rb1	**162**	*Panax ginseng* C.A.Mey. (Araliaceae)	–		C	103
Ginsenoside Rc	**163**	*Panax ginseng* C.A.Mey. (Araliaceae)	–		C	103
Ginsenoside Re	**164**	*Panax ginseng* C.A.Mey. (Araliaceae)	–		C	103
Ginsenoside Rf	**165**	*Panax ginseng* C.A.Mey. (Araliaceae)	–		C	103
Ginsenoside Rg1	**166**	*Panax ginseng* C.A.Mey. (Araliaceae)	–		C	103
Ginsenoside Rg3	**35**	*Panax ginseng* C.A.Mey. (Araliaceae)	–		C	103
Ginsenoside Rh2	**167**	*Panax ginseng* C.A.Mey. (Araliaceae)	–		C	103
Glycyrrhetinic acid	**168**		–		C	152
Hesperetin	**169**	*Citrus* sp. (Rutaceae)	–	288.8	C	93
			–	267.4	C	153
Hesperidin	**170**		–		C	93
Hirsutenone	**171**	*Alnus japonica* (Thunb.) Steud. (Betulaceae)	+	14.9	B	154
Mallotoxin	**36**	*Mallotus philippensis* (Lam.) Müll.Arg. (Euphorbiaceae)	–		B	105
Methoxsalen	**172**		–		C	93
7,8-(Methylenedioxy) flavone	**173**	*Galenia africana* L. (Aizoaceae)	–		C	69
Morin	**174**		–	111.4	C	93
Myricetin	**175**		–		C	93
Naringenin	**31**	*Citrus* sp. (Rutaceae)	+	36.5	A	93
			–	102.6	C	94
			–	102.3	C	93
			–	173.3	C	95
Naringin	**32**		–		C	93
Neohesperidin	**176**		–		C	93
Novel daphnane-type diterpenoid orthoester	**2**	*Gnidia polycephala* Gilg ex Engl. (Thymelaeaceae)	+		C	70
Ouabain	**177**		–		D	108
Oxypeucedanin	**178**	*Angelica dahurica* (Hoffm.) Benth. & Hook. f. ex Franch. & Sav. (Apiaceae)	–		A	155
Paeoniflorin	**179**	*Paeonia lactiflora* Pall. (Paeoniaceae)	–		A	156
Phorbol 12-myristate 13-acetate	**180**		–		C, D	157
Psoralen	**181**		–		C	93
Quercetin	**182**		–		C	93
Resveratrol	**183**	Fresh grape skin and red wine	–		A	129
			–		D	158
Rutin	**184**		–		C	93
Scopoletin	**185**		–		C	93
Tanshinone IIA	**186**	*Salvia miltiorrhiza* Bunge (Lamiaceae)	–		A	159
Taxifolin 3-*O*-β-d-glucopyranoside	**187**	*Rhododendron mucronulatum* Turcz. (Ericaceae)	++	9.6 (EC50)	B	160
5,7,4′-Trimethylapigenin	**188**		+	18.4–31.9	A	161
Umbelliferone	**189**		–		C	93
Yuanhuacine	**1**	*Gnidia polycephala* Gilg ex Engl. (Thymelaeaceae)	++		C	70

^a^* if provided in original study; ++ strong hERG block; + moderate hERG block; – no hERG block; A = whole-cell patch-clamp assay using HEK293 cells; B = whole-cell patch-clamp assay using CHO cells; C = two-microelectrode voltage-clamp assay using *Xenopus* oocytes; D = *ex vivo* (e.g. guinea pigs' hearts) or others.

**Fig. 4:**
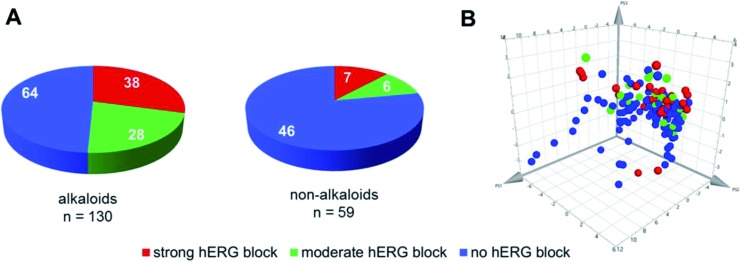
Natural compounds tested for hERG channel interactions (*n* = 189). (A) Categorization into alkaloids and non-alkaloids and previously defined activity ranges (strong, moderate, and non-blocker). (B) Chemical property space analysis. The positions are determined by the three principal components (PS1: size, PS2: aromaticity, PS3: lipophilicity), which are summarized from a large number of molecular descriptors. The colors depict strong (red), moderate (green), and non-blockers (blue).

The dataset compiled for this review covers a wide range of physicochemical properties and a diverse group of scaffolds. The hERG channel is able to interact with a diverse group of scaffolds, but in the case of natural compounds the clear majority of strong hERG channel blockers identified is concentrated among alkaloids (Fig. 4A). This trend might be related to the notion of basic nitrogen motives being responsible for high affinity hERG channel binding, and thus making this class of natural compounds in general predestined for testing. However, even for alkaloids the potency of hERG channel blockage can vary considerably within closely related compounds. A straightforward chemical space analysis *via* ChemGPS^79^ which positions compounds per size, aromaticity, and lipophilicity, is unable to separate clear clusters of blockers and non-blockers, showing compounds of all categories spread in the chemical properties plot (Fig. 4B). Notwithstanding, there is a tendency of strong and moderate blockers to be positioned in the more lipophilic region, which is in accordance with the reported positive correlation between lipophilicity and hERG channel blockage.^21^,^22^

Among further prominent structural classes tested for hERG channel blockage are flavonoids, triterpenoids, or steroids, as well as diterpenoids, stilbenes, or diarylheptanoids. However, among these scaffolds only very few strong or moderate hERG channel blockers have been identified to the moment.

### 5.1 Alkaloids

The largest group of natural compounds tested for hERG liabilities consists of alkaloids (*n* = 130). These nitrogen-containing compounds occur in characteristic plant families such as Ranunculaceae, Papaveraceae, Berberidaceae, Apocynaceae, and Rubiaceae.^49^,^67^,^72^,^80^,^81^

Due to their enormous structural diversity, establishing a classification of alkaloids is challenging. Hence, for this review, alkaloids tested for hERG channel blockage are listed alphabetically in Table 2 and the corresponding structures are grouped according to structural subclasses in Fig. S1 and S2 (ESI†). In case the alkaloid was isolated from a specific plant material, the corresponding source is given in Table 2. Otherwise, often studies were performed with purchased compounds where the natural source is not mentioned.

**Fig. 5:**
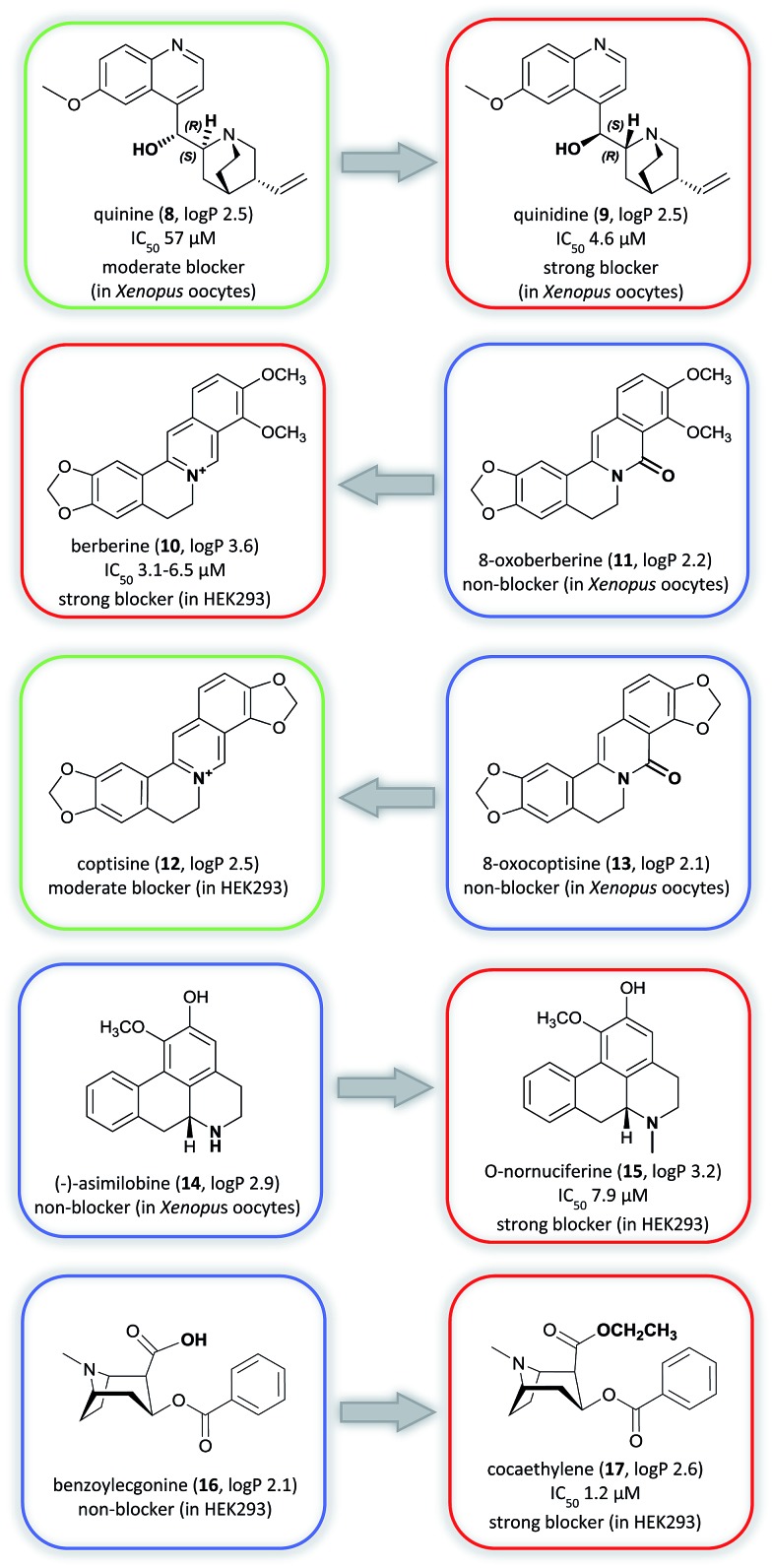
Matched molecular pairs analysis showing strong (red), moderate (green), and non-blockers (blue). The log *P* calculations were performed with Marvin (Chemaxon).

In general, alkaloid-mediated inhibition of the hERG channel was observed in almost all structural sub-classes, corroborating the important role of the basic nitrogen and hydrophobic groups,^82^ which are present throughout this diverse chemical class. However, enantioselective effects seem to play an important role in the potency of hERG channel blockage. This is underlined in the example of the well-studied diastereoisomers quinine (**8**) and quinidine (**9**) used for the treatment of malaria or arrhythmia (class Ia), respectively (Fig. 5).^83^,^84^

Both alkaloids, known as constituents of *Cinchona* sp. bark, block the hERG channel, whereby the blockage is around 12-fold lower for **8** than for **9** (voltage-clamp assay).^85^ Very recent data suggest that the interaction with the aromatic amino acid residue F656 in the S6 domain is crucial for this selectivity.^86^

Furthermore, data on structurally closely related compounds in the group of protoberberine alkaloids, *e.g.* berberine (**10**) and 8-oxoberberine (**11**), and coptisine (**12**) and 8-oxocoptisine (**13**), have shown that small changes in the electronic environment around the amine nitrogen can result in a large difference in the hERG channel blocking potency, transforming strong blockers into non-blockers (Fig. 5).^20^–^22^

Similarly, substituents that increase lipophilicity can increase hERG channel binding, such as for (–)-asimilobine (**14**) and *O*-nornuciferine (**15**), and benzoylecgonine (**16**) and cocaethylene (**17**). In both cases the addition of alkyl chains to the parent non-blocker structure resulted in a similar analog with higher lipophilicity and strong hERG channel blocking profile (Fig. 5).
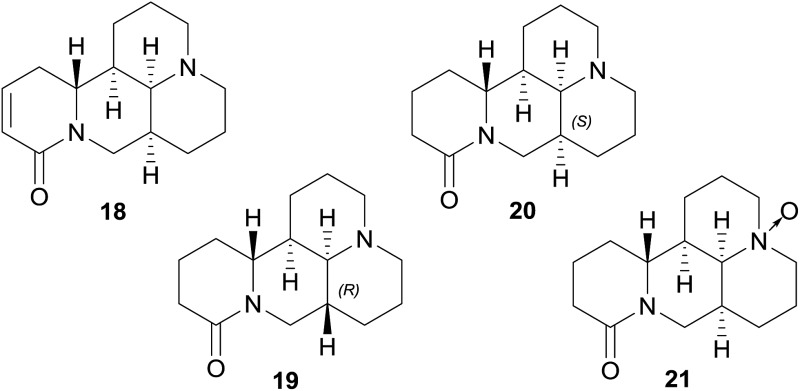


No blockage has been found among the tested tetracyclo-quinolizidine alkaloids, *i.e.* sophocarpine (**18**), sophoridine (**19**), matrine (**20**), and oxymatrine (**21**), which all lack aromatic rings in their structures.

Aromatic rings are fundamental features of the usually recognized hERG channel blocker pharmacophore, and are associated with π-stacking or hydrophobic interactions with several aromatic side chains of amino acids present in the hERG channel cavity.^17^,^19^ Additionally, chinazoline alkaloids, *i.e.* vasicine (**22**), vasicinone (**23**), and tryptanthrin (**24**),^66^,^87^–^90^ opium alkaloids, *i.e.* morphine (**25**), codeine (**26**), and (+)-salutaridine (**27**), as well as the purine alkaloids caffeine (**28**), theophylline (**29**), and theobromine (**30**) are devoid of hERG channel inhibition.^66^,^91^,^92^ 
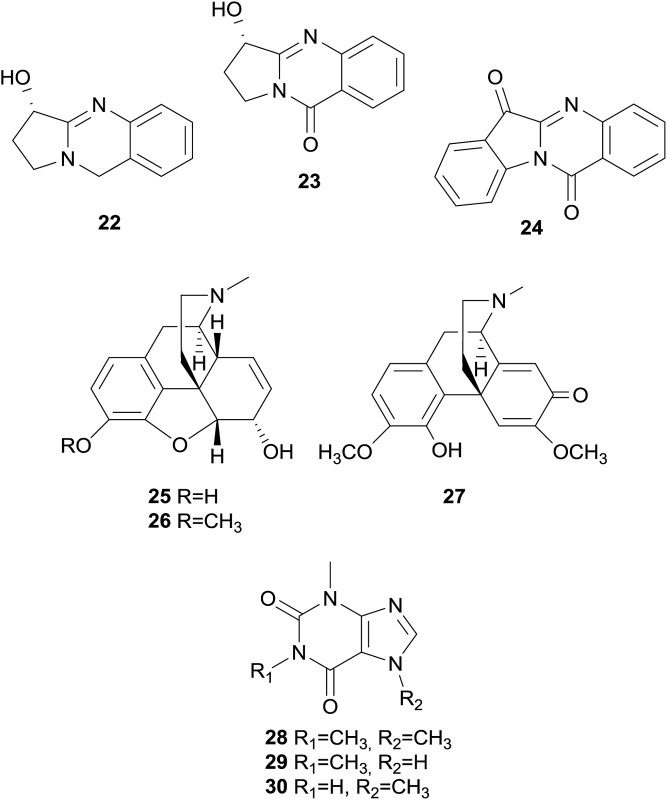


### 5.2 Non-alkaloids

The group of non-alkaloid natural compounds (*n* = 59) tested for hERG liabilities is mainly composed of flavonoids, di- and triterpenoids, as well as coumarins. Reports about strong or moderate hERG channel blockers identified among these structure classes are rather scarce, and are in accordance with the general absence of the well-recognized hERG channel blocker pharmacophore in their structures.^17^,^19^

A prominent and well-studied example is naringenin (**31**), a flavanone contained in fresh grapefruit juice.^93^ In the patch-clamp assay using HEK293 cells, this compound has shown a moderate hERG channel blocking effect (IC_50_ 36.5 μM),^93^ whereas it showed very weak or no inhibition in the voltage-clamp assay using *Xenopus* oocytes.^93^–^95^ Furthermore, grapefruit juice containing **31** and its naturally occurring glycoside naringin (**32**) were studied *in vivo*.^96^
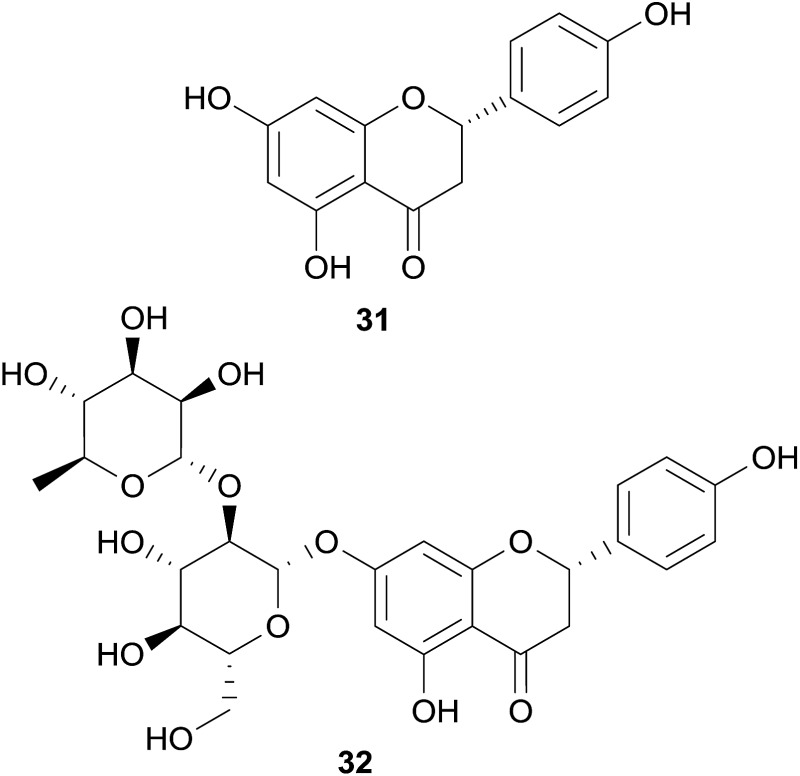


Interestingly, it was observed that a daily consumption of one liter of grapefruit juice can provoke a mild QT interval prolongation.^96^

Other examples of non-alkaloid natural compounds tested positive for hERG channel blockage are epigallocatechin-3-gallate (EGCG, **33**) present in green tea, and curcumin (**34**), present in turmeric.
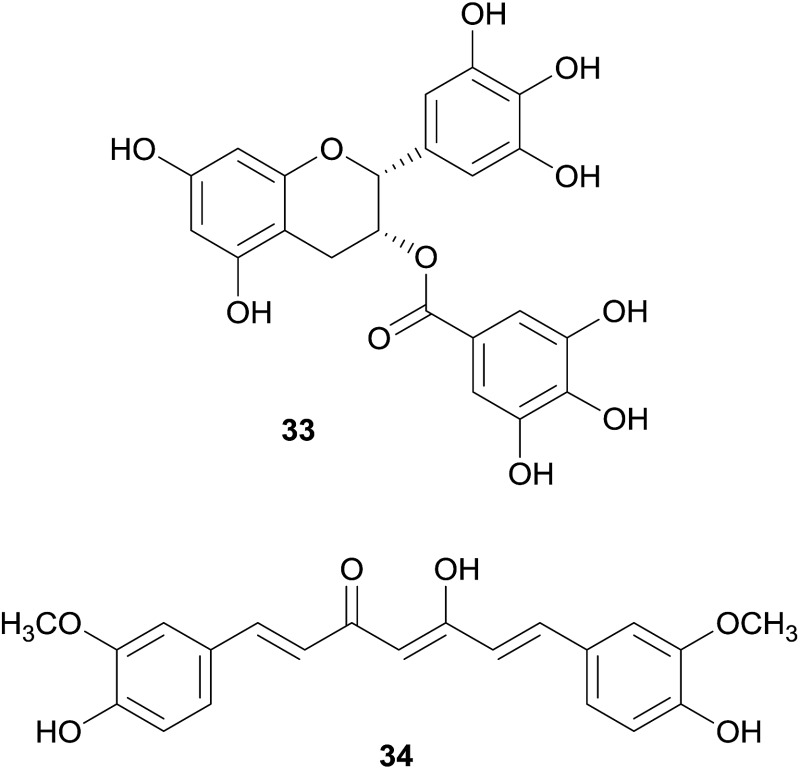


These compounds were identified as moderate and strong hERG blockers, respectively,^97^–^101^ but have also been recognized as pan-assay interference compounds, and therefore, the results should be carefully considered.^102^

### 5.3 Natural hERG channel agonists

Apart from blockers, there are also agonists of the hERG channel, which reduce the rate of channel closure and consequently shorten the AP duration and the QT interval. hERG channel activators and related antiarrhythmic effects can be beneficial in the therapy of inherited long QT syndrome where the function of the hERG channel is reduced. However, likely due to multi-channel gating properties, hERG channel agonists also have the potential of being pro-arrhythmic by extensive shortening of the QT interval.^27^

In the routine screening for hERG channel blockers, some activators have been identified by chance, and among them are also two compounds from natural origin, *i.e.* ginsenoside Rg3 (**35**) from *Panax ginseng* C.A.Mey., and mallotoxin (**36**) from *Mallotus philippensis* (Lam.) Müll.Arg. With an EC_50_ of 0.4 μM (in *Xenopus* oocytes), **35** strongly slows down the rate of channel deactivation and slightly shifts the channel opening to more negative potentials,^103^,^104^ whereas **36**, which shows these effects to a lesser extent, additionally increases the channel open probability (EC_50_s of 0.3 to 0.5 μM in CHO cells).^105^
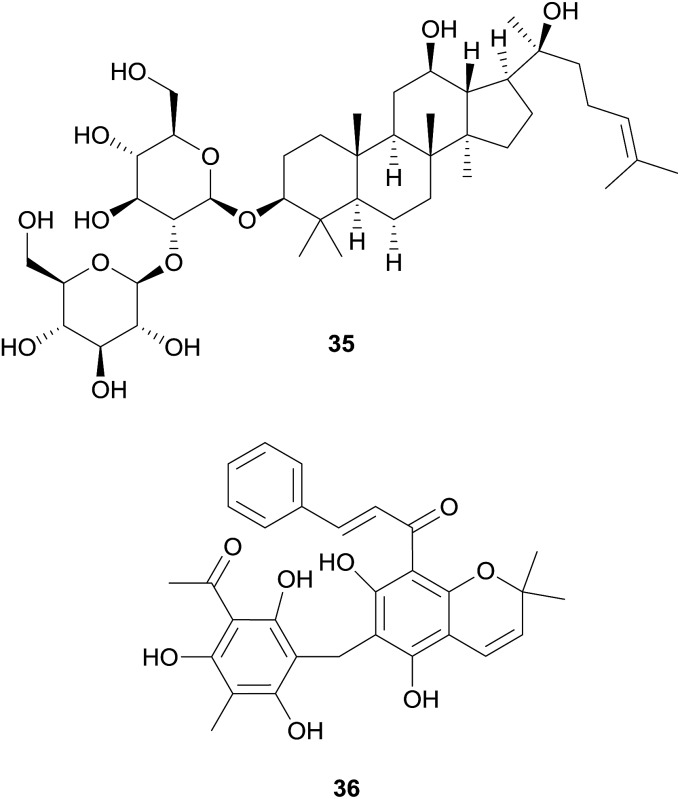


## 6 Conclusions and future perspectives

Since the connection between the hERG channel and drug-induced QT prolongation has been established, the screening for hERG channel blockage has been an important part of preclinical safety assessment. Several *in vitro* and *in vivo* models have been developed, including automated patch clamp platforms that provide the high-throughput and the high-quality data necessary for the assessment of multiple samples at an affordable and timely fashion. Harmonized guidelines have been put in place by regulatory agencies that prevented the introduction of new torsadogenic drugs to the market. Despite the effective application of this safety paradigm, an updated and comprehensive cardiac risk assessment strategy is evolving considering multi ion channel block effects.^12^ Increasing concerns on high attrition rates and the growing body of evidence on the deficiency of hERG channel blockage translatability have pushed researchers for these more complete safety assessments.

And while all new chemical entities (NCEs) must be investigated for possible hERG channel interaction, the risk portfolio of nutritionals, spices, and herbal medicines is still unsatisfying or not existing, since the word “natural” is often confused with “safe”. From our compilation of published natural compounds tested for hERG channel interaction (*n* = 189, covering the past two decades), 42%, mostly alkaloids, showed some level of *in vitro* blockage. In light of this finding, a more comprehensive investigation of natural products together with better assays and practices of preclinical cardiac safety assessment (*e.g.* multiple ion channels, mechanistic approaches) could help enlighten the potential risks of phytopharmaceuticals or even their value within drug discovery initiatives that contemplate the hERG channel as a promising target.

In the recently accomplished EU funded project ‘hERG related risk assessment of botanicals’ (PIRSES-GA-2011-295174), a consortium of nine partners from three different continents committed themselves to the evaluation of putatively hERG channel blocking properties of commonly consumed herbal remedies. The project aimed at critically assessing the hERG channel related toxicity and, thus, at improving consumer and patient safety by identifying safety liabilities of botanicals. Intriguingly, of the more than 1200 extracts probed in this endeavour, only ∼2.5% revealed a reduction of the hERG channel peak tail current by ≥30% at 100 μg mL^–1^ in the voltage-clamp assay on *Xenopus* oocytes. At a first glance, this data permits to state that commonly consumed herbal extracts bear a low risk for hERG channel-related toxicities. The very preliminary *in vitro* results of hERG channel blocking extracts however require further investigations to estimate the putative cardiotoxic profile. This refers to phytochemical investigations for the identification of the constituent/s contributing to the hERG channel blockage. On the other hand, even identified constituents with pronounced *in vitro* hERG channel blockage cannot be directly translated to an increased risk of QT prolongation. Electrophysiological studies on cardiac ion currents affecting the ventricular action potential duration as well as *in vivo* studies assessing the QT prolongation in parallel with pharmacokinetic parameters are necessary.^162^ A further aspect, which deserves special attention, is the fact that traditionally herbal remedies have not been applied to co-medicated and multi-medicated/multi-morbid patients, which renders the situation more complex. In these cases, even moderately hERG channel blocking natural compounds may contribute to or even multiply the cardiotoxic burden. This also refers to the misuse of herbal preparations as previously highlighted for ipecac,^49^ and as recently determined for the antidiarrheal drug substance loperamide.^163^,^164^

Inappropriate dosages of hERG channel blocking ingredients, which are not even labelled and quantified in the preparation, but freely accessible for consumers such as food supplements need special attention and are not *a priori* “safe” as shown in the example of lotus leaf extracts which are sold as OTC dietary weight loss supplements.^48^ In summary, the reviewed *in vitro* and *in vivo* findings on hERG channel modulation by natural products show a clear need for further investigations in this field. The relevance of identifying natural-derived hERG channel modulators is not only an issue with respect to cardiac safety liabilities of herbal remedies. There is emerging evidence for the therapeutic use of hERG channel activators, *e.g.*, for the treatment of inherited long QT syndrome by shortening the AP duration. Their relevance in newly emerging therapeutic areas, such as schizophrenia, cancer, and other co-morbidities,^5^,^28^ further underline the importance for a rapid identification of hERG channel modulators to be used as pharmacological tools and/or for the discovery and development of new lead structures.

## Supplementary Material

Supplementary information
