# Supplementary material for: Natural products modulating the hERG channel: heartaches and hope
Source: Nat Prod Rep. 2017 May 12;34(8):957–80. doi: 10.1039/c7np00014f (PMC5708533; doi:10.1039/c7np00014f)
Supplement: Supplementary file 1 [file NP-034-C7NP00014F-s001.pdf]

Electronic Supplementary Material (ESI) for Natural Product Reports.
This journal is © The Royal Society of Chemistry 2017
Please do not adjust margins

Natural Product Reports

REVIEW

Supporting Information

Received 16th February 2017,
Accepted 00th January 20xx

DOI: 10.1039/x0xx00000x

www.rsc.org/

Natural products modulating the hERG channel: heartaches and hope

Jadel M. Kratz,†[a] Ulrike Grienke,†[a] Olaf Scheel,[b] Stefan A. Mann[b] and Judith M. Rollinger*[a]

[a.] *Department of Pharmacognosy, Faculty of Life Sciences, University of Vienna, Althanstraße 14, 1090 Vienna, Austria. judith.rollinger@univie.ac.at; Fax: +43-1-4277-855255; Tel: +43-1-4277-55255*
[b.] *CytoBioscience GmbH, Nattermannallee 1, 50829 Cologne, Germany.*
† JMK and UG contributed equally.

Please do not adjust margins

Please do not adjust margins

Fig. S1 Alkaloid structures of natural compounds tested for hERG channel inhibition (grouped according to structural subclasses).

DITERPENOID ALKALOIDS

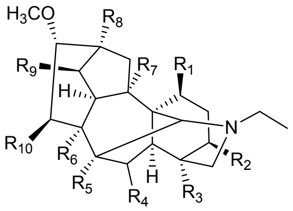

no.	R1	R2	R3	R4	R5	R6	R7	R8	R9	R10
**38**	OCH_3	H	H	H	H	OH	H	H	OH	H
**39**	OCH_3	OH	H	H	H	OH	H	H	OH	H
**66**	OH	H	CH_2OCH_3	α-OCH_3	OH	OH	H	H	OH	H
**67**	OCH_3	H	CH_2OH	α-OCH_3	OH	OH	H	H	OH	H
**68**	OH	H	CH_3	β-OH	OH	OCH_3	H	H	OH	H
**70**	OCH_3	H	H	H	H	OH	H	H	$OCOCH_3$	H
**79**	OH	H	CH_2OH	α-OCH_3	OH	OH	H	H	OCH_3	H
**86**	OCH_3	H	CH_2OCH_3	H	H	OCH_3	OH	H	OH	H
**89**	OH	H	CH_2OCH_3	H	H	OH	H	H	OH	H
**91**	OH	H	CH_3	H	H	OH	H	H	OH	H
**95**	OCH_3	H	CH_2OH	α-OCH_3	OH	OH	H	H	OCH_3	H
**106**	OH	H	CH_2OCH_3	β-OCH_3	H	OH	H	H	OH	H
**107**	OH	H	CH_2OH	β-OCH_3	H	OH	H	H	OH	H
**127**	OH	H	CH_2OCH_3	OH	H	OH	H	H	OH	H
**128**	OH	H	CH_2OCH_3	OCH_3	H	OH	H	H	OH	OH
**134**	OCH_3	H	CH_2OH	α-OCH_3	OH	OH	OH	H	OCH_3	H
**135**	OH	H	CH_2OH	α-OCH_3	OH	OH	H	H	OH	H

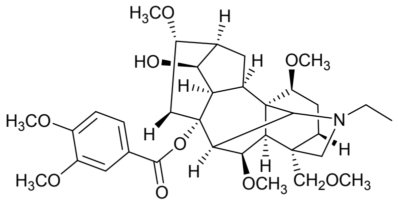

40

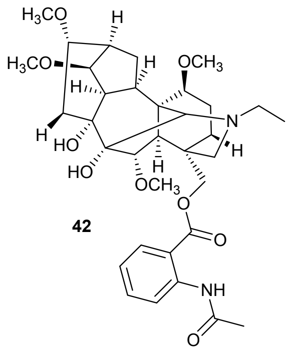

42

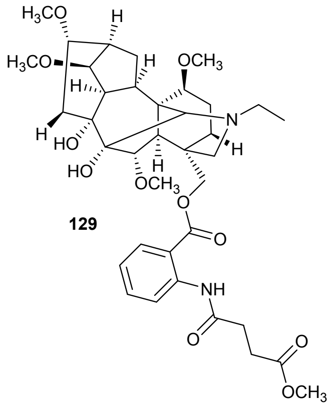

129

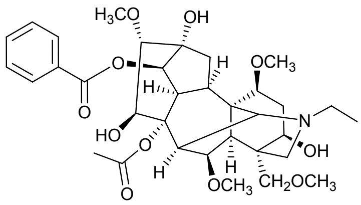

37

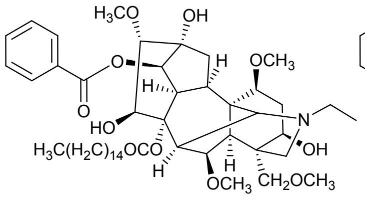

50

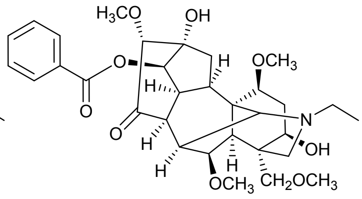

120

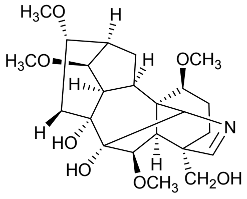

41

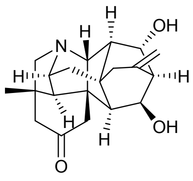

84

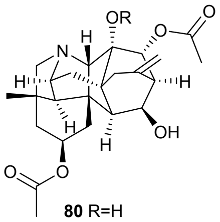

80 R=H
81 R=$OCOCH_3$

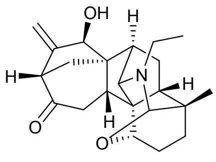

130

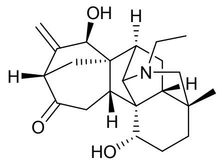

131

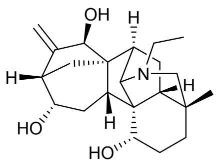

103

Please do not adjust margins

INDOLE ALKALOIDS

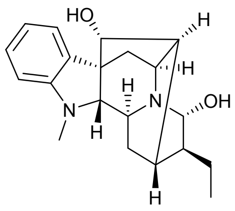

43

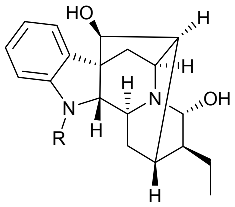

125 R=CH_3
111 R=H

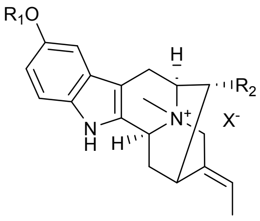

133 R_1=CH_3, R_2=CH_2OH
94 R_1=CH_3, R_2=CH_2OH
97 R_1=CH_3, R_2=COOH

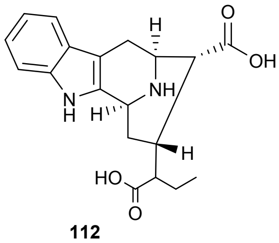

112

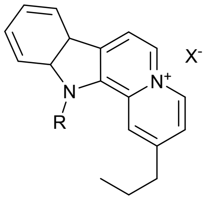

115 R=H
101 R=CH_3

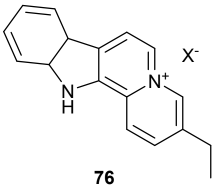

76

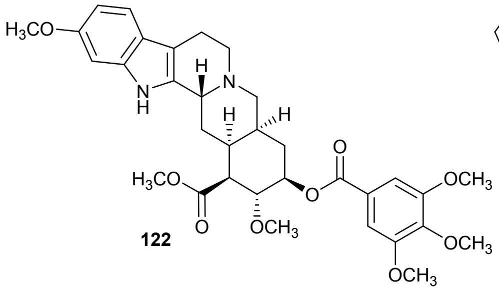

122

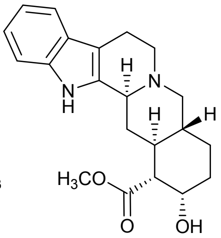

139

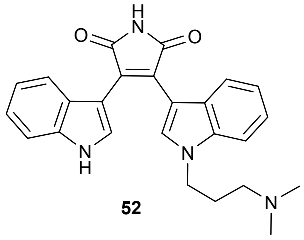

52

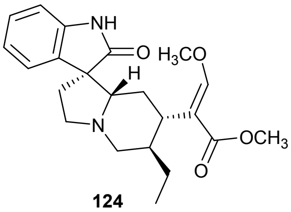

124

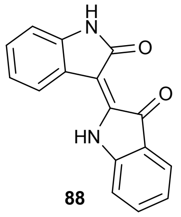

88

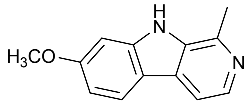

83

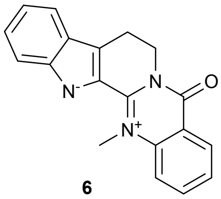

6

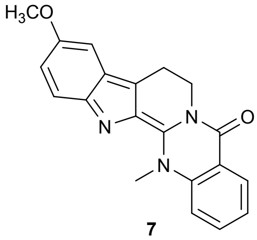

7

PROTOBERBERINE ALKALOIDS

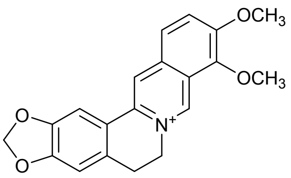

10

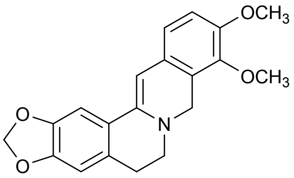

4

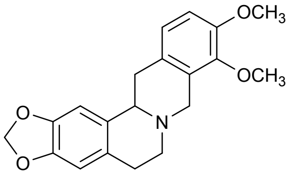

137

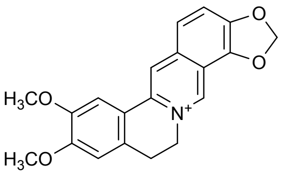

74

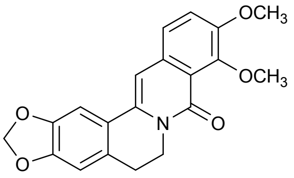

11

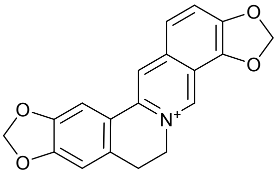

12

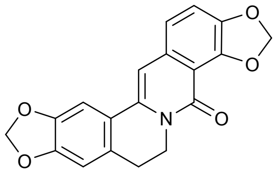

13

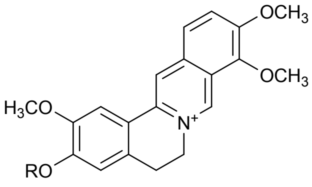

90 R=H
116 R=CH_3

Please do not adjust margins
Please do not adjust margins

QUINOLINE ALKALOIDS: APORPHINE ALKALOIDS

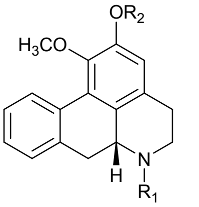

14 R_1=H, R_2=H
114 R_1=CH_3, R_2=CH_3
110 R_1=H, R_2=CH_3
15 R_1=CH_3, R_2=H

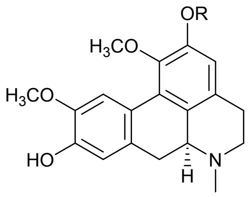

100 R=CH_3
53 R=H

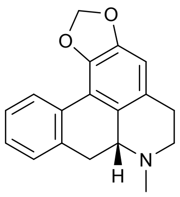

121

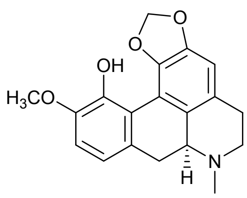

54

ISOQUINOLINE & (BIS)BENZYLISOQUINOLINE ALKALOIDS

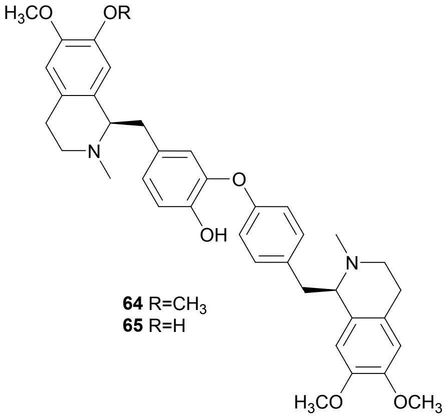

64 R=CH_3
65 R=H

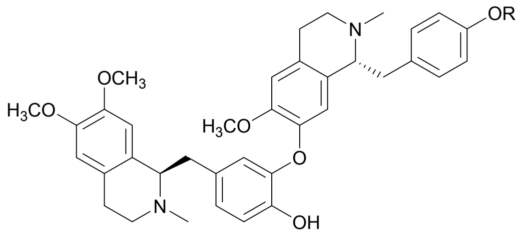

104 R=CH_3
92 R=H

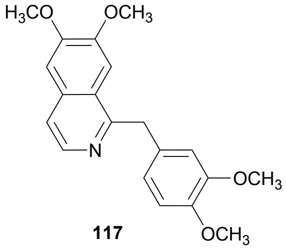

117

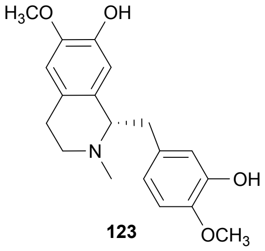

123

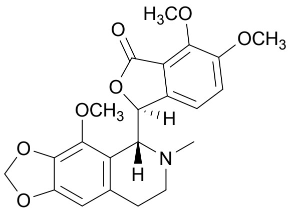

113

This journal is © The Royal Society of Chemistry 2017

BENZOPHENANTHRIDINE ALKALOIDS AND DERIVATIVES

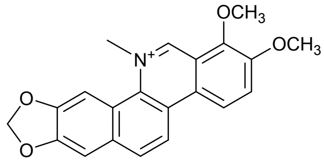

59

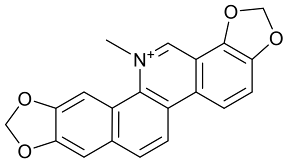

126

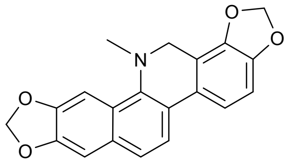

69

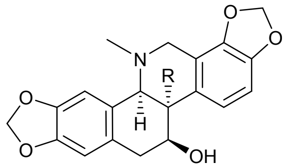

60 R=H
62 R=CH_3

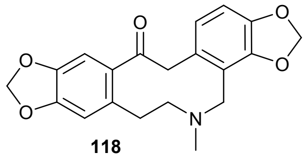

118

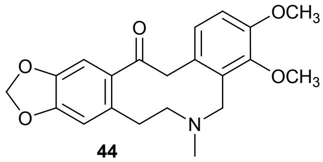

44

TROPANE ALKALOIDS

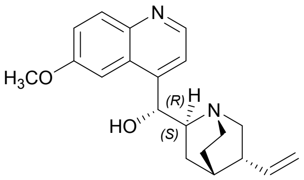

8

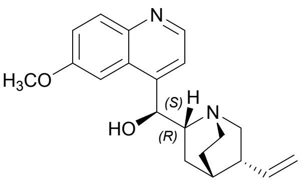

9

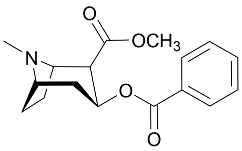

61

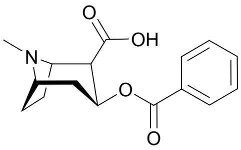

16

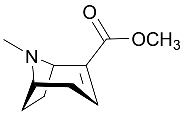

98

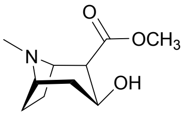

71

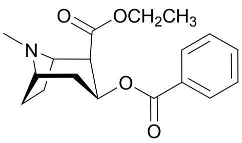

17

CHINOLIZIDIN ALKALOIDS AND DERIVATIVES

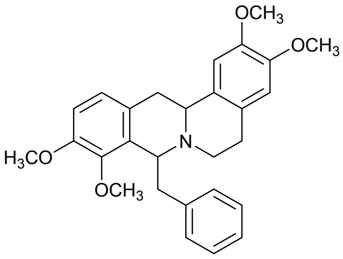

51

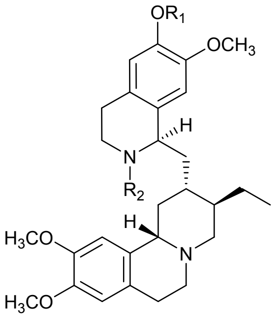

57 R_1=H, R_2=H
72 R_1=CH_3, R_2=H
99 R=CH_3, R_2=CH_3

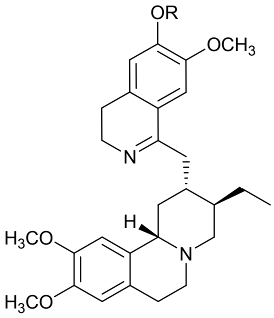

119 R=H
102 R=CH_3

Please do not adjust margins

AMARYLLIDACEAE ALKALOIDS

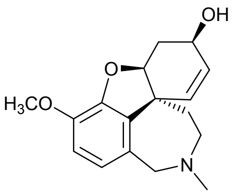

77

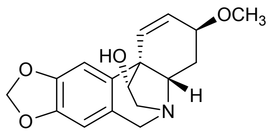

82

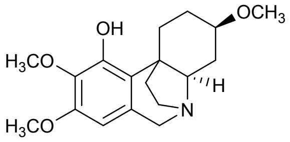

85

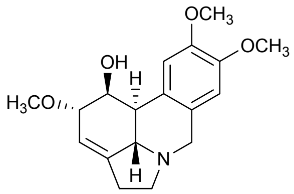

78

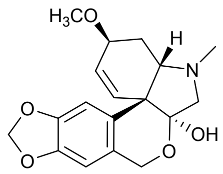

136

TETRACYCLO-QUINOLIZIDINE ALKALOIDS

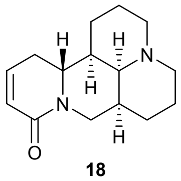

18

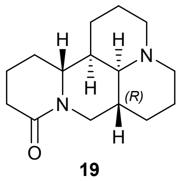

19

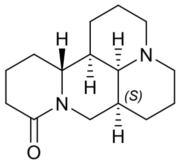

20

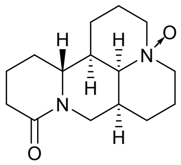

21

IBOGA ALKALOIDS

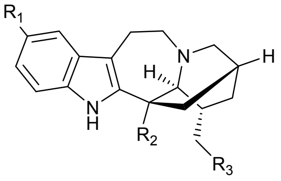

87 R_1=OCH_3 R_2=H R_3=CH_3
109 R_1=OH R_2=H R_3=CH_3
138 R_1=OCH_3 R_2=CO_2CH_3 R_3=CH_3
96 R_1=H R_2=CO_2CH_3 R_3=CH_2OCH_3

PURINE ALKALOIDS

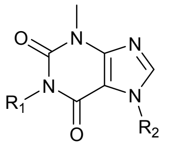

28 R_1=CH_3, R_2=CH_3
29 R_1=CH_3, R_2=H
30 R_1=H, R_2=CH_3

OPIUM ALKALOIDS

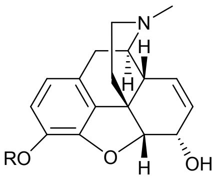

25 R=H
26 R=CH_3

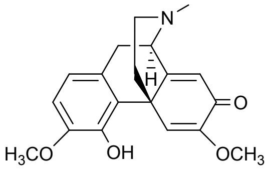

27

AMIDE & PIPERIDINE ALKALOIDS

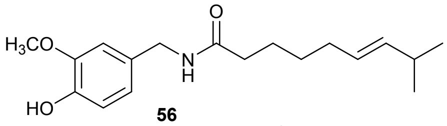

56

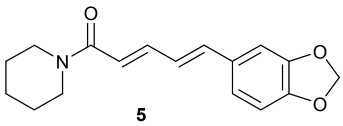

5

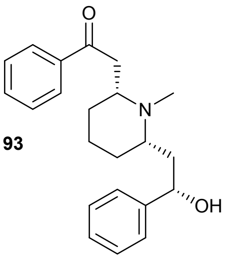

93

This journal is © The Royal Society of Chemistry 2017

Please do not adjust margins

CHINAZOLINE ALKALOIDS

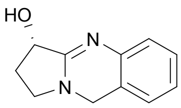

22

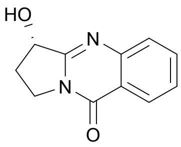

23

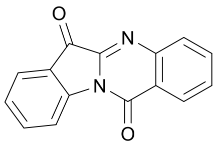

24

STEROID ALKALOIDS

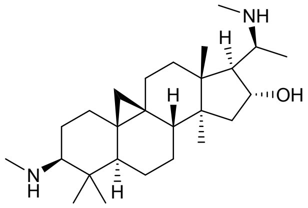

63

MARINE ALGAL TOXINS

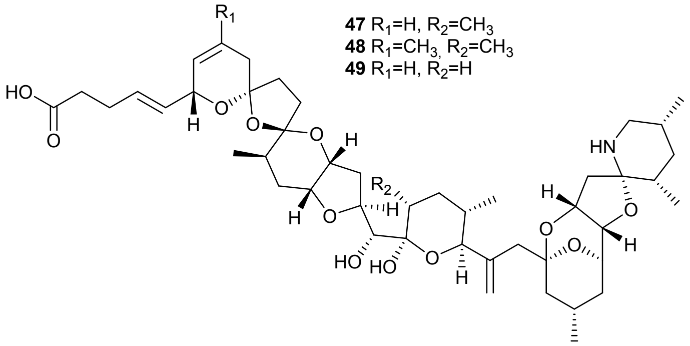

47 R_1=H, R_2=CH_3
48 R_1=CH_3, R_2=CH_3
49 R_1=H, R_2=H

MISCELLANEOUS ALKALOIDS

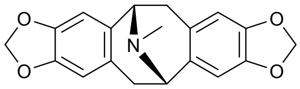

75

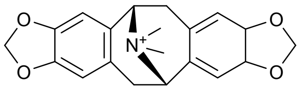

55

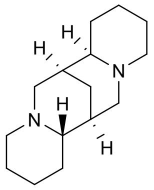

132

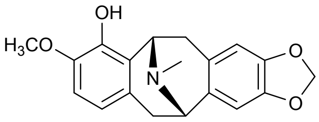

105

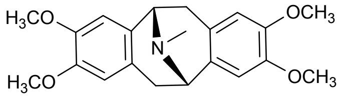

46

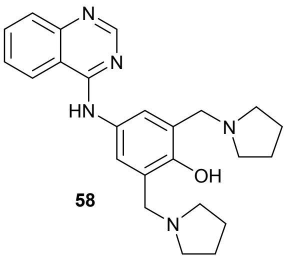

58

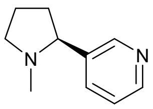

108

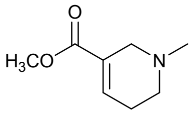

45

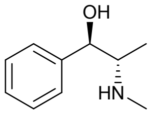

73

Please do not adjust margins

Fig. S2 **Non-alkaloid structures of natural compounds tested for hERG channel inhibition (grouped according to structural subclasses).**

FLAVONOIDS & DERIVATIVES

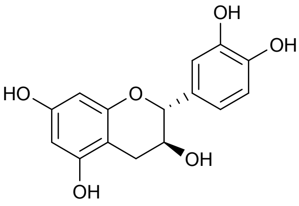

144

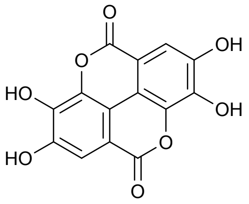

156

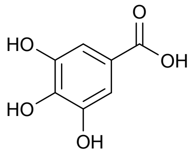

161

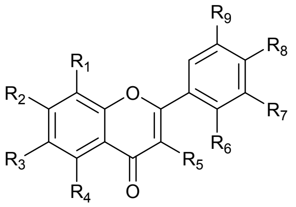

140 R_1=H, R_2=OH, R_3=H, R_4=OH, R_5=H, R_6=H, R_8=OCH_3
142 R_1=H, R_2=OH, R_3=H, R_4=OH, R_5=H, R_6=H, R_7=H, R_8=OH, R_9=H
146 R_1=H, R_2=OH, R_3=H, R_4=OH, R_5-R_6=H
152 R_1=H, R_2=OH, R_3=OCH_3, R_4=OH, R_5=H, R_6=H, R_7=H, R_8=H, R_9=H
154 R_1=H, R_2=OCH_3, R_3=OCH_3, R_4=H, R_5=H, R_6=H, R_7=H, R_8=H, R_9=H
155 R_1=OCH_3, R_2=OCH_3, R_3=H, R_4=H, R_5=H, R_6=H, R_8=H
158 R_1=H, R_2=OH, R_3=H, R_4=H, R_5=OH, R_6=H, R_7=H, R_8=OH, R_9=OH
159 R_1-R_9=H
160 R_1=H, R_2=OH, R_3=H, R_4=OH, R_5=OH, R_6=H, R_7=H, R_8=H, R_9=H
174 R_1=H, R_2=OH, R_3=H, R_4=OH, R_5=OH, R_6=OH, R_8=OH
175 R_1=H, R_2=OH, R_3=H, R_4=OH, R_5=OH, R_6=H, R_7=OH, R_8=OH, R_9=OH
182 R_1=H, R_2=OH, R_3=H, R_4=OH, R_5=OH, R_6=H, R_7=H, R_8=OH, R_9=OH
188 R_1=H, R_2=OCH_3, R_3=H, R_4=OCH_3, R_5=H, R_6=H, R_8=OCH_3

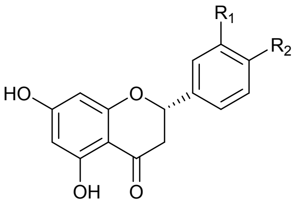

31 R_1=H, R_2=OH
169 R_1=OH, R_2=OCH_3

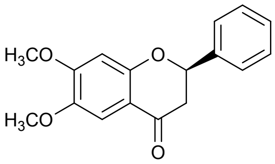

153

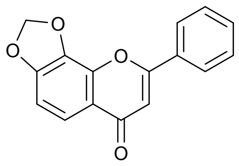

173

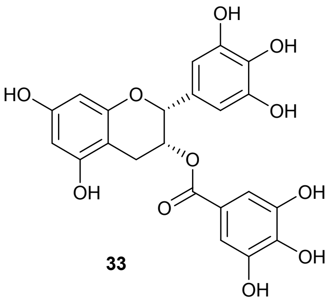

33

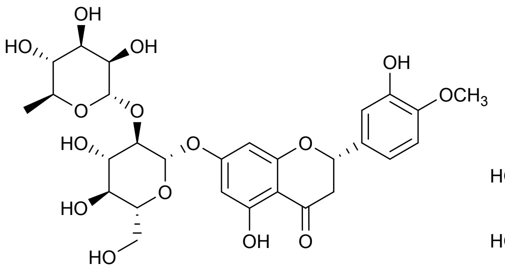

176

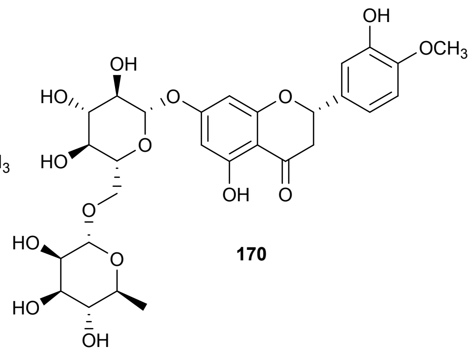

170

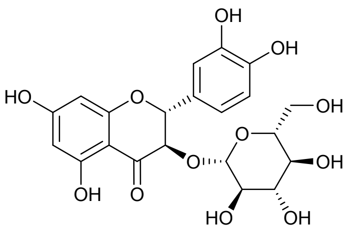

187

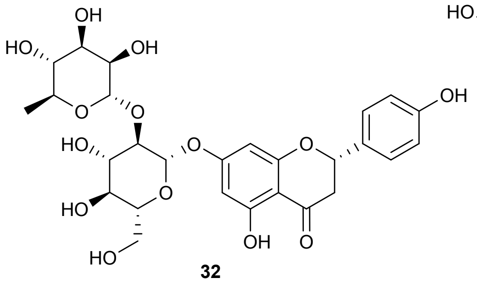

32

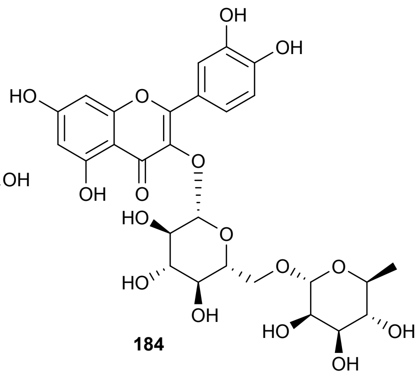

184

 This journal is © The Royal Society of Chemistry 2017

Please do not adjust margins

TRITERPENOID & STEROID DERIVATIVES

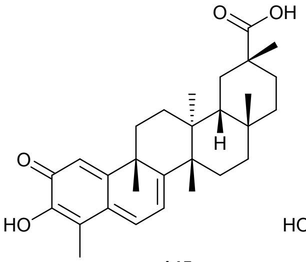

145

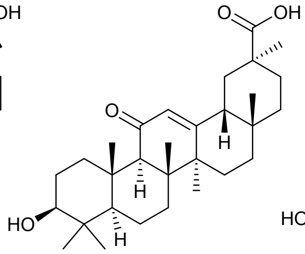

168

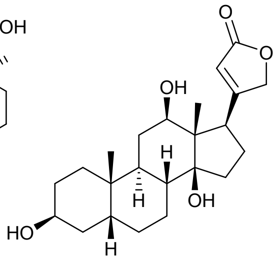

149

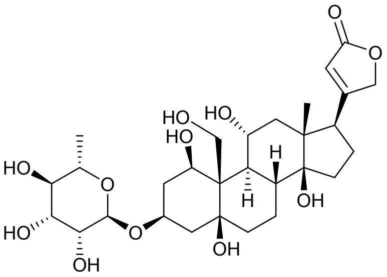

177

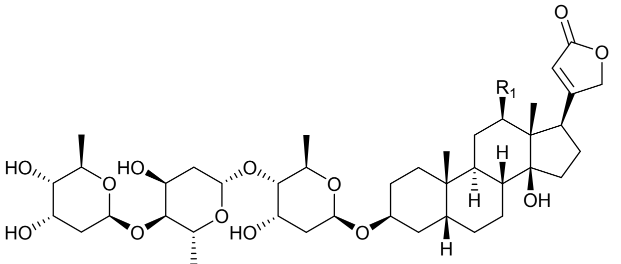

148 R_1=H
150 R_1=OH

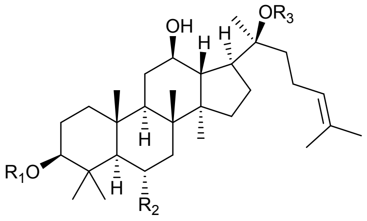

35 R_1=β-D-glc(2→1)-β-D-glc, R_2=H, R_3=H
162 R_1=β-D-glc(2→1)-β-D-glc, R_2=H, R_3=β-D-glc(6→1)-β-D-glc
163 R_1=β-D-glc(2→1)-β-D-glc, R_2=H, R_3=β-D-glc(6→1)-α-L-ara
164 R_1=H, R_2=O-β-D-glc(2→1)-α-L-rha, R_3=β-D-glc
165 R_1=H, R_2=O-β-D-glc(2→1)-β-D-glc, R_3=H
166 R_1=H, R_2=O-β-D-glc, R_3=β-D-glc
167 R_1=β-D-glc, R_2=H, R_3=H

(FURANO)COUMARINS

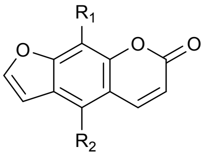

181 R_1=H, R_2=H
172 R_1=OCH_3, R_2=H
143 R_1=H, R_2=OCH_3

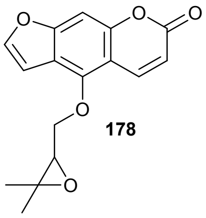

178

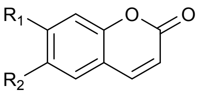

147 R_1=H, R_2=H
189 R_1=OH, R_2=H
185 R_1=OH, R_2=OCH_3
157 R_1=OCH_2CH_3, R_2=H

STILBENES & DIARYLHEPTANOIDS

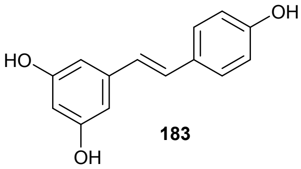

183

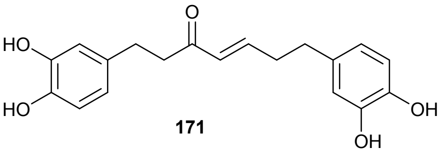

171

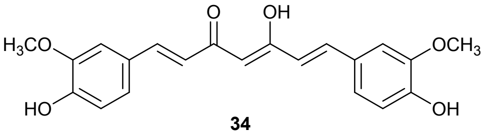

34

Please do not adjust margins

DITERPENOIDS

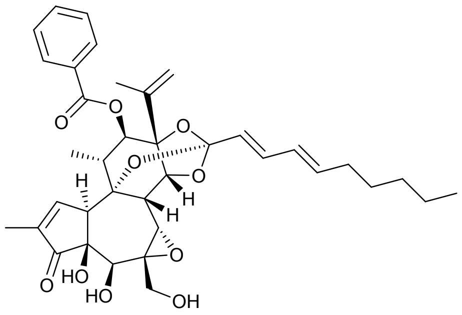

1

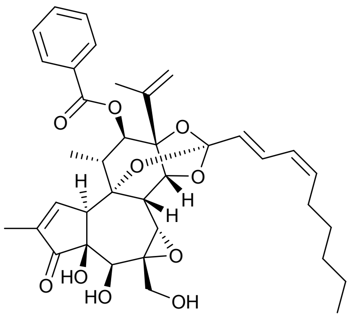

2

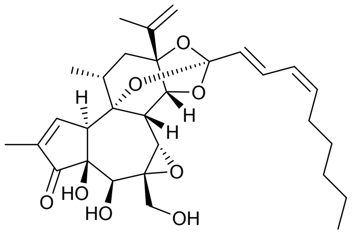

3

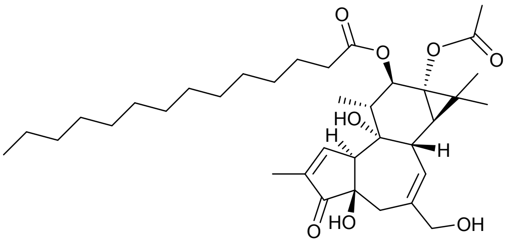

180

MISCELLANEOUS

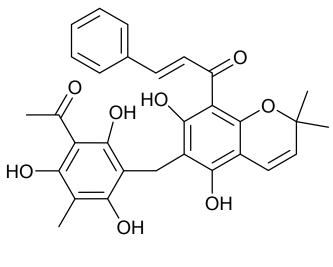

36

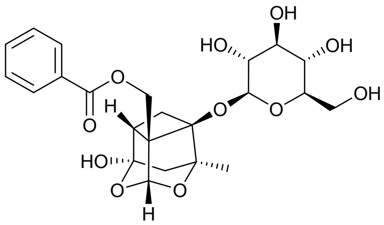

179

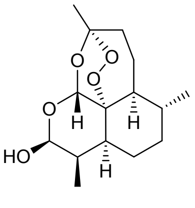

151

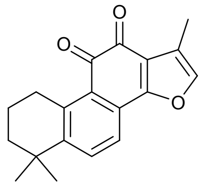

186

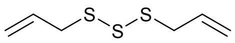

141

This journal is © The Royal Society of Chemistry 2017

Please do not adjust margins
